# Fat-1 Ameliorates Metabolic Dysfunction-Associated Fatty Liver Disease and Atherosclerosis through Promoting the Nuclear Localization of PPARα in Hamsters

**DOI:** 10.34133/research.0577

**Published:** 2025-03-06

**Authors:** Wenxi Zhang, Jiabao Guo, Guolin Miao, Jingxuan Chen, Yitong Xu, Pingping Lai, Lianxin Zhang, Yufei Han, Sin Man Lam, Guanghou Shui, Yuhui Wang, Wei Huang, Xunde Xian

**Affiliations:** ^1^ Institute of Cardiovascular Sciences, State Key Laboratory of Vascular Homeostasis and Remodeling, School of Basic Medical Sciences, Peking University, Beijing 100191, China.; ^2^ State Key Laboratory of Molecular Developmental Biology, Institute of Genetics and Developmental Biology, Chinese Academy of Sciences, Beijing 100101, China.; ^3^ LipidALL Technologies Company Limited, Changzhou 213022, Jiangsu Province, China.; ^4^ Beijing Key Laboratory of Cardiovascular Receptors Research, Peking University Third Hospital, Beijing 100191, China.

## Abstract

Fat-1, an enzyme encoded by the *fat-1* gene, is responsible for the conversion of endogenous omega-6 polyunsaturated fatty acids into omega-3 polyunsaturated fatty acids in *Caenorhabditis elegans*. To better investigate whether the expression of Fat-1 will exert a beneficial function in dyslipidemia and metabolic dysfunction-associated fatty liver disease (MAFLD), we established an adeno-associated virus 9 expressing Fat-1. We found that adeno-associated-virus-mediated expression of Fat-1 markedly reduced the levels of plasma triglycerides and total cholesterol but increased high-density lipoprotein levels in male wild-type hamsters on both chow diet and high-fat diet as well as in chow-diet-fed male LDLR^−/−^ hamsters. Fat-1 ameliorated diet-induced MAFLD in wild-type hamsters by enhancing fatty acid oxidation through the hepatic peroxisome proliferator-activated receptor α (PPARα)-dependent pathway. Mechanistically, Fat-1 increased the levels of multiple lipid derivatives as ligands for PPARα and simultaneously facilitated the nuclear localization of PPARα. Our results provide new insights into the multiple therapeutic potentials of Fat-1 to treat dyslipidemia, MAFLD, and atherosclerosis.

## Introduction

Metabolic dysfunction-associated fatty liver disease (MAFLD), formerly known as nonalcoholic fatty liver disease (NAFLD) [1], exhibits an escalating global prevalence, affecting approximately 30% of the adult population [2,3]. The nomenclature alteration from NAFLD to MAFLD is justified by its enhanced reflection of the current understanding regarding the etiology of this condition [4,5]. MAFLD encompasses a spectrum of disorders ranging from benign hepatic steatosis to more aggressive forms, including nonalcoholic steatohepatitis, cirrhosis, and hepatocellular carcinoma [6]. Obesity, type 2 diabetes mellitus, metabolic syndrome, and dyslipidemia are the top 4 risk factors for MAFLD [7,8]. A growing body of evidence suggests that MAFLD is strongly associated with the increased incidence of atherosclerotic cardiovascular disease (ASCVD), a leading cause of mortality worldwide, in which lipids such as cholesterol and triglycerides (TGs) accumulated in the liver and plasma are the primary contributors to ASCVD [9]. However, how the disturbance of lipids, especially hepatic lipids, influence atherogenic development has not been completely understood.

Omega-3 polyunsaturated fatty acids (n-3 PUFAs) are essential fatty acids for humans and can be obtained only from diet, primarily fish oil [8]. The main components of n-3 PUFAs, eicosapentaenoic acid (EPA; 20:5 n-3) and docosahexaenoic acid (DHA; 22:6 n-3), have been demonstrated to execute a wide range of beneficial effects, including lipid-lowering, anti-inflammatory, anti-arteriosclerotic, and antiplatelet properties [10]. Previous studies have shown that the levels of circulating and hepatic n-3 PUFAs and their derived lipids are lower in patients with MAFLD, and MAFLD severity is inversely associated with reduced hepatic C20 to C22 n-3 PUFAs [11]. Therefore, MAFLD may be regarded as an n-3 PUFA deficiency disease [8]. Moreover, population-based clinical studies on n-3 PUFA dietary supplements have also shown their potential to prevent liver lipid accumulation, enhance insulin sensitivity, and ameliorate metabolic dysfunction-associated steatohepatitis (MASH) in patients with MAFLD [12–15]. Consistently, Khadge et al. [16] reported that reducing the n-6/n-3 PUFA ratio and increasing n-3 PUFA intake significantly improved MAFLD. Thus, supplementation with n-3 PUFAs is a commonly used lipid-lowering strategy that reduces TG levels or TG-rich lipoproteins and has long been considered as a potential therapeutic approach for addressing abnormal liver and vascular conditions [17]. Nevertheless, the protective effects of n-3 PUFAs on more severe markers of MAFLD, such as liver injury, inflammation, and fibrosis, are still under debate. Additionally, independent studies have demonstrated that n-3 PUFA supplementation did not offer significant favorable impacts on cardiovascular events and even unexpectedly increased low-density lipoprotein cholesterol (LDL-C) levels in patients with hypertriglyceridemia [18]. Therefore, further exploration is warranted to determine the precise application scenarios for utilizing n-3 PUFAs as a potential therapy for metabolic diseases.

The *Fat-1* gene originally from *Caenorhabditis elegans* encodes an n-3 fatty acid desaturase that converts n-6 to n-3 fatty acids in the body, which is absent in mammals [19]. Different transgenic animal models overexpressing the *Fat-1* gene have been developed to study the relationship between endogenous n-3 fatty acids and various diseases such as fatty liver, atherosclerosis, arthritis, and tumors [20,21]. Notably, the Fat-1 transgene increases endogenous n-3 PUFAs to protect Fat-1 transgenic mice from high-fat diet (HFD)-induced insulin resistance, inflammation, and MAFLD. Furthermore, Fat-1 has been found to prevent high-fat-and-high-sugar-induced MAFLD through inhibiting fatty acid synthesis and the toll-like receptor 4 signaling pathway [22]. However, Liebig et al. later reported that endogenously increased n-3 PUFA levels and n-3/n-6 PUFA ratios in Fat-1 transgenic mice only transiently delayed the onset of streptozotocin/HFD-induced MASH, but failed to efficiently protect from MASH development [23,24]. Surprisingly, Wan et al. [25] discovered that endogenous n-3 PUFAs markedly elevated blood lipids in Fat-1 mice but suppressed systemic and vascular inflammation and reduced atherosclerotic lesions in ApoE^−/−^ mice. These controversial results demonstrate that the influence of Fat-1 on lipid metabolism, MAFLD, and atherosclerosis remains elusive, and whether expression of the *Fat-1* gene can be applied to treat metabolic diseases needs to be further explored.

Of note, it has widely been acknowledged that wild-type (WT) Syrian golden hamsters possess metabolic traits more similar to humans than rats and mice and are predisposed to HFD-induced hypertriglyceridemia and hypercholesterolemia. Our laboratory has previously generated a low-density-lipoprotein-receptor-deficient (LDLR^−/−^) hamster model replicating familial hypercholesterolemia (FH) [26,27], which was an ideal small rodent animal model used for studying human atherosclerosis. In the present study, to better comprehensively investigate the impact of Fat-1 on circulating and hepatic lipid metabolism, MAFLD, and atherosclerosis in different hamster models, we constructed an adeno-associated virus 9 (AAV9) vector to express Fat-1 in both WT and LDLR^−/−^ hamsters, aiming to evaluate the vital role of Fat-1 in spontaneous and HFD-induced MAFLD and atherosclerosis, and then assess the feasibility of AAV9 expressing Fat-1 (AAV9-Fat-1) as a gene therapy approach with potential clinical applications to treat human metabolic diseases.

## Results

### Expression of Fat-1 generates favorable plasma lipid profiles in WT hamsters on CD and HFD feeding

**Fig. 1.**
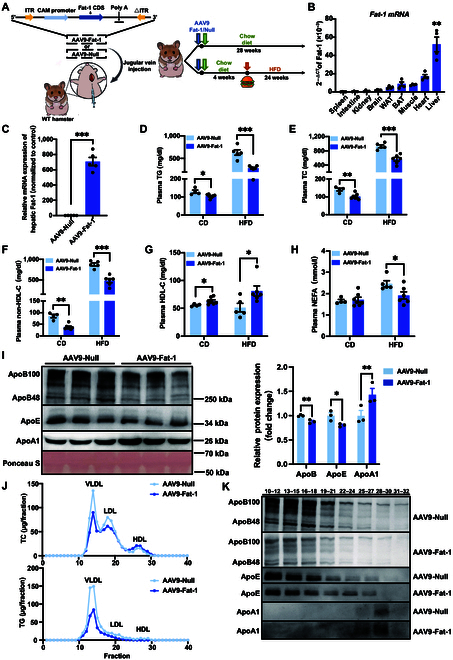
Expression of Fat-1 generates favorable plasma lipid profiles in wild-type (WT) hamsters on chow and high-fat diet (HFD) feeding. (A) Schematic showing the construction and delivery of adeno-associated virus 9 expressing Fat-1 (AAV9-Fat-1), and the experimental design. (B) Fat-1 distribution in different tissues of chow diet (CD)-fed WT hamsters on day 14 after AAV-Fat-1 injection (*n* = 5/group). **P < 0.01 versus spleen. (C) Measurement of Fat-1 messenger RNA (mRNA) expression in livers from WT hamsters on day 14 after injection with AAV9-Null or AAV9-Fat-1 (*n* = 5/group). ***P < 0.001. (D to H) Analysis of fasting plasma triglyceride (TG) (D), total cholesterol (TC) (E), non-high-density lipoprotein cholesterol (non-HDL-C) (F), high-density lipoprotein cholesterol (HDL-C) (G), and nonesterified fatty acid (NEFA) (H) levels of CD- and HFD-fed WT hamsters treated with AAV9-Null or AAV9-Fat-1 (*n* = 4 to 7/group). *P < 0.05; **P < 0.01; ***P < 0.001. (I) Representative images of Western blots showing plasma apolipoproteins (ApoB, ApoE, and ApoA1) from HFD-fed WT hamsters with or without Fat-1 expression and quantification (*n* = 3/group). *P < 0.05 and **P < 0.01. (J) Fast protein liquid chromatography (FPLC) analysis of TG and TC distribution from pooled plasma in AAV9-Null- and AAV9-Fat-1-treated WT hamsters (*n* = 5 and 6/group). (K) Representative Western blots of ApoB, ApoE, and ApoA1 in different fractions described in (J) (*n* = 5 and 6/group). All data are expressed as mean ± standard error of the mean (SEM). Statistical significance was determined by the Student *t* test. *P < 0.05; **P < 0.01; ***P < 0.001. ApoB, apolipoprotein B; ApoE, apolipoprotein E; ApoA1, apolipoprotein A1; LDL, low-density lipoprotein; VLDL, very low-density lipoprotein; ITR, inverted terminal repeat; CAM, calmodulin; CDS, coding sequence; WAT, white adipose tissue; BAT, brown adipose tissue.

In the present study, to investigate the impact of Fat-1 on lipid profiles and the efficacy and the safety of gene therapy for MAFLD and atherosclerosis, we first constructed an AAV9 vector expressing Fat-1 or null and then injected it via the jugular vein into WT hamsters at a dose of 1 × 10^13^ vg/kg, and then the animals were fed a standard chow diet (CD) for 28 weeks or an HFD containing 20% fat and 0.5% cholesterol for 24 weeks following 4-week CD (Fig. 1A). We found that the messenger RNA (mRNA) levels of Fat-1 were significantly increased in different organs of WT hamsters on week 4 after virus administration, including the spleen, intestine, kidney, brain, muscle, heart, and liver, among which the liver had the highest Fat-1 mRNA expression level, suggesting that the AAV9 vector effectively expressed Fat-1 in our hamster model (Fig. 1B). However, no detectable Fat-1 was observed in the control group treated with AAV9-Null (Fig. 1C).

To explore the influence of AAV9-Fat-1 treatment on plasma lipid profiles under physiological or over nutrient conditions, we measured the concentrations of plasma TG, total cholesterol (TC), non-high-density lipoprotein cholesterol (non-HDL-C), high-density lipoprotein cholesterol (HDL-C), and nonesterified fatty acid (NEFA) in WT hamsters fed on CD or HFD, respectively. We observed that administration of AAV9-Fat-1 significantly decreased the levels of plasma TG, TC, and non-HDL-C but increased plasma HDL-C in WT hamsters compared to those in the AAV9-Null-treated groups on CD and HFD feeding (Fig. 1D to G). Fat-1 also reduced the plasma NEFA levels in HFD-fed WT hamsters, but there was no significant difference in plasma NEFA levels between the 2 groups on CD (Fig. 1H). Next, we detected important apolipoproteins (Apo) in plasma. In comparison with AAV9-Null-treated hamsters, the levels of ApoB100 and ApoE were markedly decreased, while the level of ApoA1 was significantly increased in AAV9-Fat-1-treated WT hamsters on CD (Fig. S1A) and HFD (Fig. 1I). Furthermore, fast protein liquid chromatography was performed to analyze the distribution of plasma lipids. Consistent with the results of plasma lipids, cholesterol contents in the very low-density lipoprotein (VLDL) and low-density lipoprotein (LDL) fractions were notably decreased, and the TG concentration was also reduced in the VLDL fraction; however, the concentration of cholesterol carried on HDL particles was markedly increased after AAV9-Fat-1 administration under both CD and HFD conditions (Fig. S1B and C and Fig. 1J and K). Furthermore, we determined the blood glucose levels in CD-fed hamsters and found that Fat-1 did not affect the glucose levels (Fig. S1D). Meanwhile, glucose tolerance test and insulin tolerance test experiments were performed, showing that Fat-1 did not have a significant effect on glucose tolerance and insulin sensitivity (Fig. S1E and F). Together, these findings demonstrate that treatment with AAV9-Fat-1 can effectively generate favorable plasma lipid profiles of WT hamsters under CD and HFD conditions.

### Fat-1 ameliorates hepatic steatosis in HFD-fed WT hamsters

Since Fat-1 executed beneficial function on hyperlipidemia, it was rational for us to investigate the effect of the expression of Fat-1 on MAFLD in WT hamsters. Although Fat-1 reduced basal plasma TG and TC levels, CD-fed WT hamsters did not have hepatic steatosis (Fig. S1G to J). Thus, we focused only on the pathological changes in the livers of WT hamsters under HFD conditions.

**Fig. 2.**
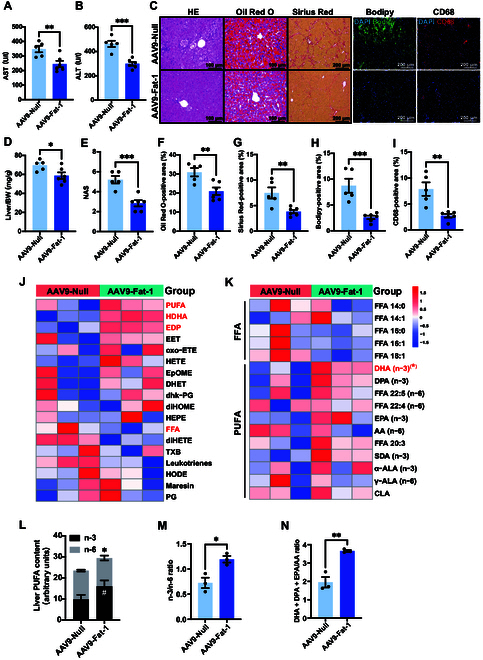
Fat-1 ameliorates hepatic steatosis in HFD-fed WT hamsters. (A and B) Plasma aspartate aminotransferase (AST) (A) and alanine aminotransferase (ALT) (B) were determined from HFD-fed WT hamsters after treatment with AAV9-Null (*n* = 5) or AAV9-Fat-1 (*n* = 6). **P < 0.01 and ***P < 0.001. (C) The representative images of hematoxylin and eosin (HE), Oil Red O, and Picrosirius Red staining; Bodipy and CD68 staining by immunofluorescence of the liver sections from the animals described in (A) (*n* = 5 and 6/group). (D) The ratio of liver weight and body weight from the animals described in (A) (*n* = 5 and 6/group). *P < 0.05. (E) Quantification of the nonalcoholic fatty liver disease (NAFLD) activity score (NAS) of AAV9-Null- and AAV9-Fat-1-treated WT hamsters on HFD (*n* = 5 and 6/group). ***P < 0.001. (F to I) Quantitative analysis of Oil Red O (F), Picrosirius Red (G), Bodipy (H), and CD68 (I) staining area in (C) using ImageJ. **P < 0.01 and ***P < 0.001. (J) Lipidomic analyses of the livers from HFD-fed WT hamsters after treatment with AAV9-Null or AAV9-Fat-1 (*n* = 3/group). (K) The heatmap of changed free fatty acid (FFAs) and polyunsaturated fatty acids (PUFAs) in the liver lipidomics from the 2 groups described in (J) (*n* = 3/group). *P < 0.05. (L) The contents and composition of PUFAs in liver samples from the indicated animals (*n* = 3/group). “*”indicates differences between the summed abundance of n-3 and n-6 PUFAs in the liver (*P < 0.05), and “#” indicates differences in n-3 PUFA content in the liver (^#^*P* < 0.05). (M and N) The ratio of n-3 to n-6 (M) and of (DHA + DPA + EPA)/AA (N) in livers from the indicated hamsters (*n* = 3/group). The n-6:n-3 fatty-acid ratio is given by (18:2 n-6 + 20:4 n-6 + 22:4 n-6 + 22:5 n-6):(18:3 n-3 + 20:5 n-3 + 22:5 n-3 + 22:6 n-3). Data are presented as mean ± SEM. Statistical significance was determined by the Student *t* test. *P < 0.05; **P < 0.01; ***P < 0.001; ^#^*P* < 0.05. DHA, docosahexaenoic acid (22:6 n-3); DPA, docosapentaenoic acid (22:5 n-3); EPA, eicosapentaenoic acid (20:5 n-3); AA, arachidonic acid; SDA, stearidonic acid (18:4 n-3); ALA, α-linolenic acid (18:3 n-3); DAPI, 4′,6-diamidino-2-phenylindole; BW, body weight; HDHA, hydroxydocosahexaenoic acid; EDP, epoxydocosapentaenoic acid; EET, epoxyeicosatrienoic acid; oxo-ETE, oxo-eicosatetraenoic acid; HETE, hydroxyeicosatetraenoic acid; EpOME, epoxyoctadecenoic acid; DHET, dihydroxyeicosatrienoic acid; dhk-PG, dehydroketo prostaglandin; diHOME, dihydroxyoctadecenoic acid; HEPE, hydroxyeicosapentaenoic acid; diHETE, dihydroxyeicosatetraenoic acid; TXB, thromboxane B; HODE, hydroxyoctadecadienoic acid; PG, prostaglandin; CLA, conjugated linoleic acid.

Firstly, we monitored liver function at the endpoints of experiments in each group and found that plasma aspartate aminotransferase (AST) and alanine aminotransferase (ALT) levels were significantly decreased in the AAV9-Fat-1-treated group compared to the control group (Fig. 2A and B) without affecting other blood biochemical parameters (Fig. S2A to O), clearly indicating that AAV9-Fat-1 alleviated liver injury induced by HFD. Moreover, WT hamsters treated with AAV9-Fat-1 had a lower liver/body weight ratio after being fed HFD for 24 weeks (Fig. 2D); however, the administration of Fat-1 had no effect on the body weight or the weights and ratios of other organs, including the heart, kidney, adipose tissue, and brain (Fig. S3A to I). Secondly, we investigated the morphological changes in the livers of WT hamsters mentioned above. Hematoxylin and eosin (HE) staining of livers revealed prominent morphological alterations in the control group, characterized by lipid droplet accumulation, fibrosis, and enhanced inflammatory response (Fig. 2C). Remarkably, administration of AAV9-Fat-1 significantly ameliorated these phenotypes with a reduction in the NAFLD activity score (NAS) (Fig. 2E). Moreover, the livers stained with Oil Red O and Bodipy exhibited a higher degree of hepatic steatosis in the control group (Fig. 2F and H), whereas the AAV9-Fat-1-treated group showed apparently reduced lipid deposition. Additionally, Fat-1 significantly relieved liver fibrosis and macrophage infiltration in the liver sections, as demonstrated by Picrosirius Red staining and CD68 immunofluorescence staining, respectively (Fig. 2G and I). In conclusion, AAV9-Fat-1 can protect against liver injury, lipid deposition, and inflammation caused by HFD in WT hamsters.

Based on the previous finding that MAFLD severity is associated with hepatic PUFAs [8], we conducted targeted lipidomics to assess the hepatic PUFAs and their derived bioactive lipid autacoids in HFD-fed WT hamsters. Compared to the control hamsters, AAV9-Fat-1-treated hamsters exhibited elevated levels of hepatic PUFA, epoxydocosapentaenoic acid (EDP), and hydroxydocosahexaenoic acid (HDHA) with significantly reduced contents of free fatty acid (FFA). Notably, EDP and HDHA were prominent bioactive lipid autacoids derived from DHA (Fig. 2J). As expected, we found that Fat-1 expression noticeably increased the DHA content in the livers of WT hamsters fed HFD while reducing the levels of saturated and monounsaturated fatty acids (Fig. 2K). Quantitative analysis revealed that treatment with Fat-1 significantly augmented the overall abundance of PUFAs in the liver while concomitantly elevating the proportion of n-3 PUFAs (Fig. 2L). Moreover, there was a notable increase in both the n-3/n-6 fatty acid ratio and the ratio between the key constituents of n-3 PUFAs (DHA, DPA, and EPA) and the primary component of n-6 PUFA arachidonic acid (AA) within the liver tissue (Fig. 2M and N). Our results presented here demonstrate that the potential of Fat-1 to improve MAFLD severity induced by an HFD in WT hamsters is probably attributable to an increase in DHA and its derivative HDHA levels in the livers.

### Fat-1 activates the PPARα signaling pathway in HFD-fed WT hamsters

**Fig. 3.**
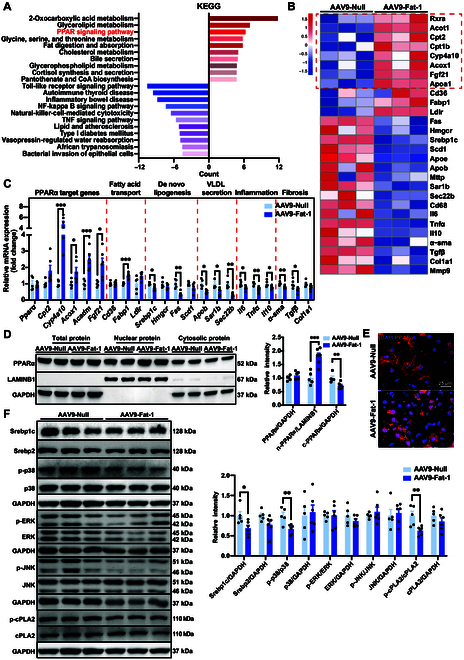
Fat-1 activates the peroxisome proliferator-activated receptor α (PPARα) signaling pathway in HFD-fed WT hamsters. (A) Kyoto Encyclopedia of Genes and Genomes (KEGG) pathway enrichment analysis of the top 10 enriched up-regulated pathways and the top 10 enriched down-regulated pathways of the transcriptome data in the livers of HFD-fed WT hamsters after treatment with AAV9-Null (*n* = 5) or AAV9-Fat-1(*n* = 6). (B) Gene set enrichment analysis (GSEA) of PPARα targets, fatty acid β-oxidation, transport, de novo lipid synthesis, VLDL secretion, inflammation, and fibrosis (*n* = 3/group). (C) The mRNA expression levels of genes involved in the processes described in (B) were quantified using real-time polymerase chain reaction (PCR) in the liver samples from the indicated groups described in (A) (*n* = 5 and 6/group). *P < 0.05; **P < 0.01; ***P < 0.001. (D) The protein level of total PPARα, nuclear PPARα, and cytoplasmic PPARα in the liver of animals described in (A) and quantification. The signals of total PPARα and cytoplasmic PPARα were normalized to glyceraldehyde 3-phosphate dehydrogenase (GAPDH), while nuclear PPARα signal was normalized to Lamin B1 (LAMINB1). **P < 0.01 and ***P < 0.001. (E) Immunofluorescence staining of liver PPARα from the hamsters described in (A). (F) Representative blot images of hepatic expression of sterol regulatory element-binding protein 1c (Srebp1c), sterol regulatory element-binding protein 2 (Srebp2), phosphorylated p38 mitogen-activated protein kinase (p-p38), p38, phosphorylated extracellular signal-regulated kinase (p-ERK), extracellular signal-regulated kinase (ERK), phosphorylated c-Jun N-terminal kinase (p-JNK), c-Jun N-terminal kinase (JNK), phosphorylated cytosolic phospholipase A2 (p-cPLA2), and cytosolic phospholipase A2 (cPLA2) proteins in the liver of animals described in (A) and quantification. The levels of Srebp1c, Srebp2, p38, ERK, JNK, and cPLA2 were normalized to GAPDH. Densitometry of p-p38, p-ERK, and p-JNK signals was normalized to total p38, ERK, and JNK, respectively. *P < 0.05 and **P < 0.01. Data are presented as mean ± SEM. Statistical significance was determined by the Student *t* test. *P < 0.05; **P < 0.01; ***P < 0.001. CoA, coenzyme A; NF, nuclear factor; TNF, tumor necrosis factor.

To further explore the molecular mechanism underlying the therapeutic effects of AAV9-Fat-1 treatment on MAFLD, we performed transcriptomics analysis on the livers from WT hamsters fed HFD. The enriched Kyoto Encyclopedia of Genes and Genomes pathways show that AAV9-Fat-1 significantly up-regulates lipid digestion and absorption, cholesterol metabolism, bile secretion, and peroxisome proliferator-activated receptor (PPAR) signaling pathways. Conversely, lipid and atherosclerosis as well as many inflammation- and infection-related pathways such as nuclear factor kappa B and tumor necrosis factor signaling are markedly down-regulated (Fig. 3A). To gain a more comprehensive understanding of the pathways regulated by Fat-1, we performed gene set enrichment analysis, which unveiled enrichment of peroxisome proliferator-activated receptor α (PPARα) target genes in the Fat-1 group, while genes associated with de novo lipid (DNL) synthesis, VLDL secretion, inflammation, and fibrosis were significantly down-regulated (Fig. 3B). Subsequently, we validated these findings through quantitative real-time polymerase chain reaction experiments and observed that AAV9-Fat-1 treatment significantly up-regulated the mRNA levels of PPARα target genes such as *Cyp4a10*, *Acox1*, *Acadm*, and *Fgf21* as well as *Fabp1* involved in lipid transport in the livers of WT hamsters fed with HFD. These results suggest that Fat-1 promotes fatty acid transportation to mitochondria and then enhances fatty acid β-oxidation (FAO). Meanwhile, Fat-1 treatment markedly reduced the expression levels of genes associated with the DNL synthesis pathway, including *Srebp1c* and *Fas*, as well as genes involved in VLDL secretion, such as *ApoB*, *Sar1b*, and *Sec22b*. Furthermore, consistent with histopathological observations, Fat-1 expression significantly down-regulated the mRNA levels of *Il6*, *Tnfα*, and *Il10*, the important markers of inflammatory response as well as fibrosis-associated *α-sma* and *Tgfβ* (Fig. 3C).

PPARα serves as the central transcriptional regulator of FAO; however, Fat-1 intervention did not exert any influence on the mRNA and total protein levels of PPARα. Since nuclear localization is an essential prerequisite for transcriptional regulators to modulate gene expression, we further investigated the nuclear localization of PPARα. Interestingly, Fat-1 significantly augmented the nuclear protein level of PPARα while reducing its cytoplasmic counterpart, consequently leading to a substantial increase in the nuclear-to-cytoplasmic ratio (Fig. 3D and E). Moreover, treatment with Fat-1 markedly diminished Srebp1c protein abundance and suppressed p38 mitogen-activated protein kinases (MAPKs) and cytosolic phospholipase A2 (cPLA2) phosphorylation (Fig. 3F). In summary, Fat-1 facilitates the translocation of PPARα into the nucleus, thereby activating PPARα-mediated FAO while concurrently suppressing DNL synthesis and inflammation in the liver.

### Fat-1 promotes the nuclear localization of PPARα to alleviate lipid accumulation in HepG2 cells

**Fig. 4.**
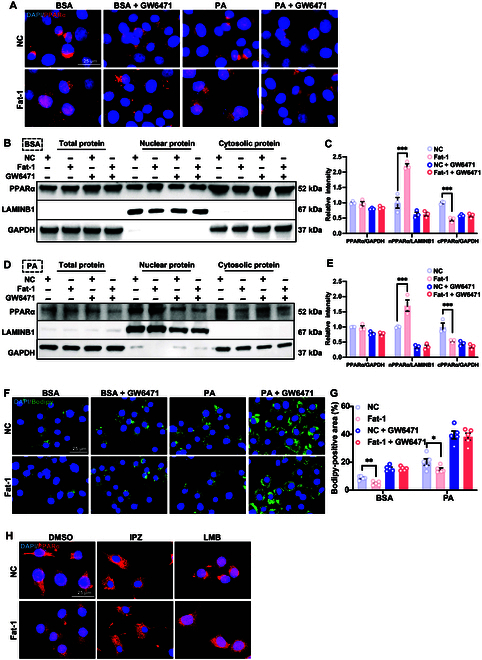
Fat-1 promotes the nuclear localization of PPARα to alleviate lipid accumulation in HepG2 cells. HepG2 cells were transfected with the empty vector (negative control, NC) or the Fat-1 plasmid for 6 h and then incubated with dimethyl sulfoxide (DMSO; 10 μM) or GW6471 (10 μM) for 6 h prior to treatment with bovine serum albumin (BSA; 300 μM) or palmitic acid (PA; 300 mM) for 12 h. (A) Immunofluorescence staining of PPARα in HepG2 cells (representative images are shown for each condition from one of 3 biologically independent experiments). (B and C) Total, nuclear, and cytoplasmic protein levels of PPARα after BSA treatment (300 μM) for 12 h in HepG2 cells (*n* = 3/group) (B). The results of protein quantification are presented on the right side (C). ***P < 0.001. (D and E) Total, nuclear, and cytoplasmic protein levels of PPARα after PA treatment (300 μM) for 12 h in HepG2 cells (*n* = 3/group) (D). The results of protein quantification are presented on the right side (E). ***P < 0.001. (F and G) Immunofluorescence staining of Bodipy in HepG2 cells (Representative images are shown for each condition from 1 of 3 biologically independent experiments.) (F). The results of fluorescence intensity quantification are presented on the right side (G). *P < 0.05 and **P < 0.01. (H) Immunofluorescence of PPARα in HepG2 cells transfected with the NC or Fat-1 plasmid in the presence and absence of importazole (IPZ; 10 μM) or leptomycin B (LMB; 10 ng/μl). Data are presented as mean ± SEM. Statistical significance was determined by the Student *t* test. *P < 0.05; **P < 0.01; ***P < 0.001.

To confirm the direct regulation of nuclear localization of PPARα by Fat-1 in vitro, we transfected HepG2 cells with the empty vector (negative control) or the Fat-1 plasmid and performed immunofluorescence staining. The results demonstrated that under both bovine serum albumin (300 μM) or palmitic acid stimulation conditions, Fat-1 significantly enhanced the nuclear localization of PPARα. However, this promoting effect was abolished upon treatment with GW6471, a specific inhibitor of PPARα (Fig. 4A). Consistently, Western blot analysis revealed that Fat-1 specifically increased the nuclear content of PPARα protein without affecting its overall expression level (Fig. 4B to E). Furthermore, Bodipy staining revealed that Fat-1 effectively attenuated lipid accumulation in HepG2 cells under both bovine serum albumin and palmitic acid conditions, which was abrogated upon administration of GW6471 (Fig. 4F and G), indicating that the lipid-lowering effect of Fat-1 depends on PPARα.

Given the dynamic nature of the nucleoplasmic shuttling process of PPARα, we sought to ascertain the impact of Fat-1 on the nuclear import and export process of PPARα. To this end, we employed importazole and leptomycin B, the inhibitors of nuclear import and the nuclear export inhibitor, respectively. We found that Fat-1 treatment promoted the nuclear import capacity of PPARα, increasing the nuclear levels of PPARα; however, Fat-1 did not alter the nuclear export of PPARα (Fig. 4H), which was consistent with the previous observations that the entry of PPAR into the nucleus depends on a ligand’s presence [28]. Herein, we postulated that as PPARα’s ligands, increased endogenous PUFAs generated by Fat-1 could bind to PPARα in the cytoplasm and then activated its nuclear translocation to regulate gene transcription and subsequently mitigate lipid accumulation.

### Blockade of PPARα activity hampers the protective effect of Fat-1 on MAFLD in HFD-fed WT hamsters

**Fig. 5.**
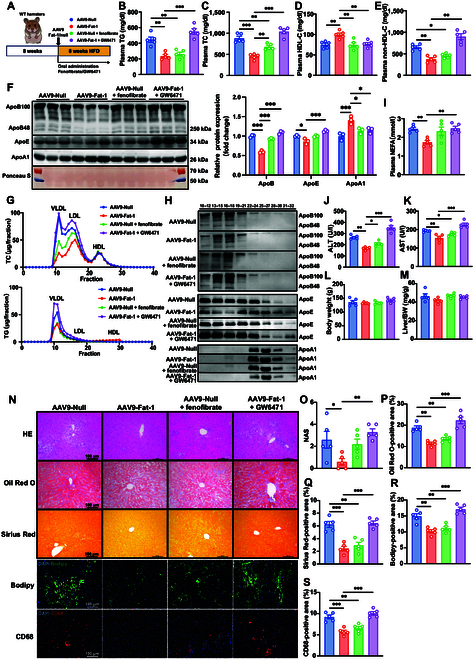
Blockade of PPARα activity hampers the protective effect of Fat-1 on metabolic dysfunction-associated fatty liver disease (MAFLD) in HFD-fed WT hamsters. (A) Schematic representation of the in vivo experiments showing that AAV9-Null or AAV9-Fat-1 was injected into the jugular vein of 8-week-old WT hamsters. AAV9-Null-treated hamsters received an oral administration of the PPARα agonist fenofibrate (100 mg/kg/d) or the control vehicle (0.5% CMCNa), while AAV9-Fat-1-treated hamsters was orally administered with the PPARα inhibitor GW6471 (1 mg/kg/d) or the control vehicle (0.5% carboxymethylcellulose sodium [CMCNa]). Samples were collected after 8 weeks on an HFD. (B to E) Analysis of the levels of fasting plasma TG (B), TC (C), HDL-C (D), and non-HDL-C (E) in the animals described in (A) (*n* = 5/group). *P < 0.05; **P < 0.01; ***P < 0.001. (F) Representative images of Western blots showing plasma ApoB, ApoE, and ApoA1 from the hamsters described in (A) and quantification (*n* = 3/group). *P < 0.05 and ***P < 0.001. (G) FPLC analysis of TG and TC distribution from the pooled plasma in hamsters described in (A) (*n* = 5/group). (H) Representative Western blots showing ApoB, ApoE, and ApoA1 in different fractions as described in (G). (I) Analysis of the fasting plasma NEFA levels of the animals described in (A) (*n* = 5/group). **P < 0.01. (J and K) Plasma ALT (J) and AST (K) were determined from the animals described in (A) (*n* = 5/group). *P < 0.05; **P < 0.01; ***P < 0.001. (L and M) Body weight and liver weight-to-body weight ratio from the animals described in (A) (*n* = 5/group). (N) The representative images of HE, Oil Red O, and Picrosirius Red, Bodipy, and CD68 stainings in the liver sections from the animals described in (A). (O to S) Quantitative analysis of NAS (O) and Oil Red O (P), Picrosirius Red (Q), Bodipy (R), and CD68 (S) staining areas in (N) using ImageJ (*n* = 5/group). *P < 0.05; **P < 0.01; ***P < 0.001. All data are expressed as mean ± SEM. Statistical significance was determined by 2-way analysis of variance (ANOVA). *P < 0.05; **P < 0.01; ***P < 0.001.

To further investigate whether the beneficial effect of Fat-1 depends on PPARα activity in vivo, 8-week-old WT hamsters were intravenously injected with AAV9-Fat-1 via the jugular vein and concurrently administered the PPARα inhibitor GW6471 (1 mg/kg/d) orally. Meanwhile, we comprehensively compared the therapeutic efficacy of Fat-1 and the most common PPARα agonist fenofibrate (Fig. 5A).

Consistent with previous findings, treatment with Fat-1 significantly reduced the levels of TG, TC, non-HDL-C, and NEFA while significantly increasing HDL-C levels in the AAV9-Null group. However, the beneficial effects of Fat-1 were nullified by the administration of GW6471, a PPARα inhibitor. The PPARα agonist fenofibrate effectively decreased plasma TG, TC, and non-HDL-C levels but had no significant impact on HDL-C and NEFA levels. Furthermore, compared to fenofibrate treatment alone, AAV9-Fat-1 notably elevated plasma HDL-C levels (Fig. 5B to E and I). Western blot analysis revealed that Fat-1 down-regulated the levels of ApoB and ApoE proteins while up-regulating ApoA1 protein in plasma; however, these effects were abolished when PPARα was inhibited by GW6471. Additionally, fenofibrate treatment did not significantly affect plasma apolipoprotein levels compared to the control group (Fig. 5F). Fast protein liquid chromatography results demonstrated that AAV9-Fat-1 substantially improved lipid distribution, in line with previous findings, by reducing cholesterol and TG content in VLDL fraction as well as cholesterol content in LDL fraction; however, this improvement was negated by co-administration of the PPARα inhibitor GW6471 (Fig. 5G and H). The effect of Fat-1 on the enhancement of plasma HDL-C and NEFA levels, as well as plasma apoprotein levels, surpasses that of fenofibrate.

We subsequently assessed the hepatic function markers ALT and AST at the study endpoints in each experimental group. Consistent with our previous findings, treatment with Fat-1 significantly attenuated ALT and AST levels, and this protective effect was abrogated upon inhibition of PPARα. Conversely, fenofibrate exhibited a significant reduction in AST levels; however, its impact on ALT levels was notably weaker compared to that observed with Fat-1 treatment (Fig. 5J and K). Furthermore, we evaluated the body weight and liver-to-body ratio among all 4 hamster groups but did not observe any statistically significant differences (Fig. 5L and M). We further investigated the morphological changes in the liver of WT hamsters. HE staining revealed that GW6471 reversed the effect of Fat-1 on ameliorating the severity of MAFLD, while fenofibrate did not significantly affect the liver NAS (Fig. 5N and O). Oil Red O and Bodipy staining demonstrated that both fenofibrate and Fat-1 treatment reduced hepatic lipid accumulation, but the protective effect of Fat-1 on lipid accumulation was abolished upon inhibition of PPARα (Fig. 5N, P, and R). Sirius Red staining and CD68 immunofluorescence staining consistently showed that PPARα inhibition reversed the improvement exerted by Fat-1 on liver fibrosis and inflammation (Fig. 5N, Q, and S). These findings suggest that Fat-1 exerts its effects on lipid profile and MAFLD through dependence on the PPARα signaling pathway. Although fenofibrate can also reduce blood lipids and improve fatty liver, it is less effective than Fat-1 in improving HDL-C levels and NEFA levels, as well as optimizing plasma apolipoprotein levels and lipoprotein distribution.

### Fat-1 expression decreases plasma TG and TC levels but increases plasma the n-3 and n-6 PUFA concentration in LDLR^−/−^ hamsters on CD

**Fig. 6.**
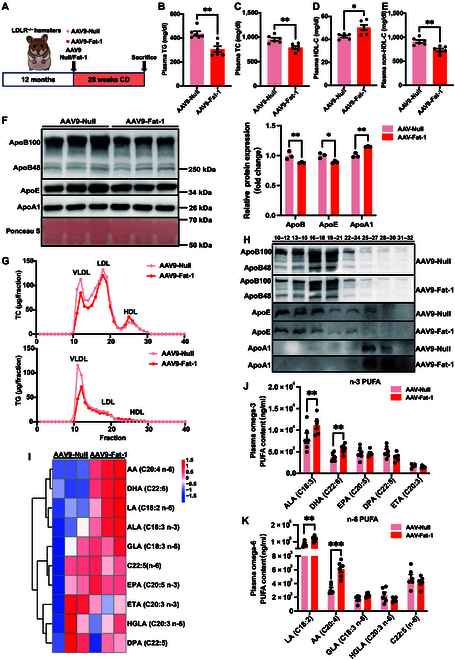
Fat-1 expression decreases plasma TG and TC levels but increases plasma n-3 and n-6 PUFA concentration in LDLR^−/−^ hamsters on CD. (A) Schematic representation of 12-month-old male LDLR^−/−^ hamsters fed a CD for 28 weeks (*n* = 6/group). (B to E) Fasting plasma TG (A), TC (B), HDL-C (C), and non-HDL-C (D) levels of CD-fed LDLR^−/−^ hamsters after treatment with AAV9-Null or AAV9-Fat-1 (*n* = 6). *P < 0.05 and **P < 0.01. (F) Representative images of Western blots showing plasma apolipoproteins (ApoB, ApoE, and ApoA1) from CD-fed LDLR^−/−^ hamsters with or without Fat-1 expression and quantification (*n* = 3/group). *P < 0.05 and **P < 0.01. (G) FPLC analysis of TG and TC distribution from pooled plasma in AAV9-Null- and AAV9-Fat-1-treated LDLR^−/−^ hamsters on CD (*n* = 6). (H) Representative Western blots of ApoB, ApoE, and ApoA1 in different fractions described in (F) (*n* = 6). (I) Targeted lipidomic analysis of plasma samples from CD-fed LDLR^−/−^ hamsters treated with AAV9-Null or AAV9-Fat-1. (J and K) The contents of plasma n-3 PUFAs (I) and n-6 PUFAs (J) from the indicated animals (*n* = 6). **P < 0.01 and ***P < 0.001. Data are presented as mean ± SEM. Statistical significance was determined by the Student *t* test. *P < 0.05; **P < 0.01; ***P < 0.001. GLA, gamma-linolenic acid; HGLA, high gamma-linolenic acid.

Our laboratory previously generated LDLR^−/−^ hamsters, a well-established model for hyperlipidemia, steatohepatitis, and atherosclerosis [26]. Accordingly, we studied the impact of AAV9-Fat-1 intervention on spontaneous hyperlipidemia and steatohepatitis in LDLR^−/−^ hamsters under CD condition. AAV9-Null or AAV9-Fat-1 was injected via the jugular vein into 12-month-old male LDLR^−/−^ hamsters at a dose of 1 × 10^13^ vg/kg, followed by CD feeding for 28 weeks (Fig. 6A). Similar to the findings observed in WT hamsters, AAV9-Fat-1 treatment markedly decreased the levels of plasma TG, TC, and non-HDL-C while elevating plasma HDL-C levels (Fig. 6B to E). Consistently, the plasma ApoB and ApoE levels were also decreased, accompanied by an increase in ApoA1 levels in AAV9-Fat-1-treated LDLR^−/−^ hamsters compared to that in the control group (Fig. 6F). In the meantime, AAV9-Fat-1 treatment effectively corrected the abnormal plasma lipoprotein profile observed in LDLR^−/−^ hamsters, showing a reduction in both cholesterol and TG contents in the VLDL and LDL fractions accompanied by the elevated concentration of cholesterol distributed in the HDL fraction, which was similar to the observations reported in the WT group (Fig. 6G). Furthermore, Western blot analysis indicated an obvious decrease in ApoB and ApoE levels within the VLDL and LDL fractions, along with an elevation of ApoA1 levels within the HDL fractions from the AAV9-Fat-1-treated group (Fig. 6H).

Next, to elucidate the impact of AAV9-Fat-1 on plasma PUFA profiles in LDLR^−/−^ hamsters, we applied target lipidomics to analyze the composition of plasma PUFAs in the indicated animals. The results showed that the treatment with AAV9-Fat-1 significantly augmented levels of n-3 PUFAs, including ALA (C18:3) and DHA (C22:6), as well as markedly elevated 2 classes of n-6 PUFAs—LA (C18:2) and AA (C20:4)—in circulation (Fig. 6I to K), which were distinct from those observed in WT hamsters. These findings reveal that AAV9-Fat-1 plays a crucial role in reducing blood lipids, improving plasma the lipid profile, and increasing plasma PUFA levels in CD-fed LDLR^−/−^ hamsters.

### Fat-1 elicits an abundance of n-3 fatty acids in the livers of CD-fed LDLR^−/−^ hamsters

**Fig. 7.**
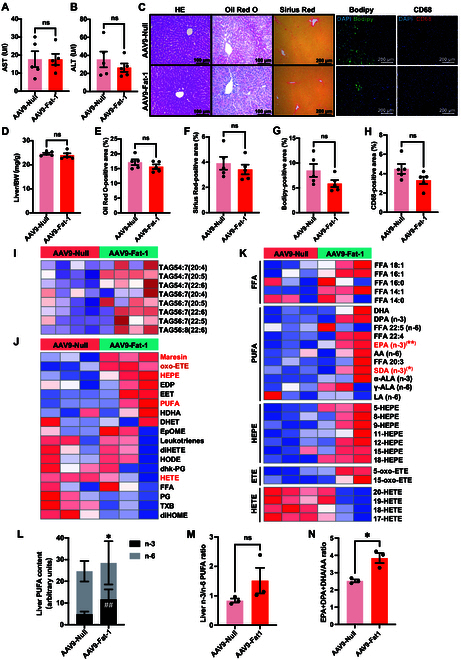
Fat-1 elicits an abundance of n-3 fatty acids in the livers of CD-fed LDLR^−/−^ hamsters. (A and B) Plasma AST (A) and ALT (B) were determined from CD-fed LDLR^−/−^ hamsters treated with AAV9-Null or AAV9-Fat-1 (*n* = 5/group). ns, not significant. (C) The representative images of HE, Oil Red O, Picrosirius Red, Bodipy, and CD68 staining by immunofluorescence of the liver sections from the animals described in (A) (*n* = 5/group). (D) The ratio of liver weight and body weight from the animals described in (A) (*n* = 5/group). ns, not significant. (E to H) Quantitative analysis of Oil Red O (E), Picrosirius Red (F), Bodipy (G), and CD68 (H) staining area in (C) using ImageJ. ns, not significant. (I) Lipidomic analysis of liver samples from CD-fed LDLR^−/−^ hamsters treated with AAV9-Null or AAV9-Fat-1. The heatmap of changed triacylglycerols (TAGs) in the liver lipidomics from the 2 groups described in (A) (*n* = 4/group). (J) Unsupervised clustering of indicated 2 groups based on the quantification of 18 lipids (in rows) in liver samples. (K) The heatmap of changed FFAs, PUFAs, HEPEs, epoxyeicosatrienoic acids (ETEs), and HETEs in the liver lipidomics from the 2 groups described in (J) (*n* = 3/group). *P < 0.05 and **P < 0.01. (L) The contents and composition of PUFAs in liver samples from the indicated animals (*n* = 3/group). “*” indicates differences between the summed abundance of n-3 and n-6 PUFAs in the liver (*P < 0.05), and “#” indicates differences in n-3 PUFA content in the liver (^##^*P* < 0.01). (M and N) The ratio of n-3 to n-6 (M) and of (EPA + DPA + DHA)/AA (N) in livers from the indicated hamsters (*n* = 3). The n-6:n-3 fatty-acid ratio is given by (18:2 n-6 + 20:4 n-6 + 22:4 n-6 + 22:5 n-6):(18:3 n-3 + 20:5 n-3 + 22:5 n-3 + 22:6 n-3). Data are presented as mean ± SEM. Statistical significance was determined by the Student *t* test. ns, not significant.

Since hyperlipidemia has been considered as a risk factor for MAFLD, we investigated the effect of Fat-1 on liver injury and hepatic lipid metabolism in CD-fed LDLR^−/−^ hamsters. The results of plasma AST and ALT levels, as well as liver/body weight ratio, showed no significant differences between the 2 groups (Fig. 7A, B, and D). Histological analysis of the liver using HE staining revealed no apparent morphological changes in the livers of AAV9-Null- and AAV9-Fat-1-treated LDLR^−/−^ hamsters (Fig. 7C). Additionally, Oil Red O staining and Bodipy immunofluorescence staining demonstrated that AAV9-Fat-1 could moderately attenuate lipid deposition in the liver (Fig. 7C), but the quantitative analysis did not reach statistical difference between the 2 groups (Fig. 7E and G). Similarly, Picrosirius Red staining and CD68 immunofluorescence staining also showed a trend of decrease in fibrosis or inflammation in the AAV9-Fat-1-treated group, respectively (Fig. 7F and H).

In order to investigate how Fat-1 impacts the composition of liver PUFAs in LDLR^−/−^ hamsters under CD conditions, we performed lipidomic analysis of the liver and found that AAV9-Fat-1-treated LDLR^−/−^ hamsters exhibited a significantly higher abundance of triacylglycerols containing n-3 and n-6 PUFAs compared to the control group (Fig. 7I). Furthermore, targeted lipidomic analysis revealed an increase in maresin (MaR), oxo-eicosatetraenoic acid, hydroxyeicosapentaenoic acid (HEPE), and PUFA in the livers of AAV9-Fat-1-treated LDLR^−/−^ hamsters. Specifically, MaRs were derived from DHA, while oxo-eicosatetraenoic acid and HEPE were derived from EPA. Additionally, Fat-1 treatment significantly decreased the levels of hydroxyeicosatetraenoic acid, a metabolite derived from n-6 PUFA AA, in the livers compared to the control group. Consistently, there was a significant elevation in EPA and stearidonic acid contents in the livers of the AAV9-Fat-1-treated LDLR^−/−^ hamsters. Despite Fat-1 having no significant effect on the total amount of FFA, it elicited the content of monounsaturated fatty acids in FFA (Fig. 7J and K). Consistent with previous findings, Fat-1 treatment increased the content of PUFAs in the liver of CD-fed LDLR^−/−^ hamsters, particularly enhancing the proportion of n-3 PUFAs (Fig. 7L). Interestingly, although no statistical difference was observed, an increasing trend of hepatic n-3/n-6 ratio was noted in LDLR^−/−^ hamsters treated with AAV9-Fat-1 (Fig. 7M). However, the ratio of EPA, DPA, and DHA to AA was markedly elevated in the AAV9-Fat-1-treated group (Fig. 7N), indicating a favorable profile of PUFAs in the livers of CD-fed LDLR^−/−^ hamsters. Our results demonstrate that Fat-1 promotes higher levels of EPA and EPA-derived oxylipins to maintain liver homeostasis in CD-fed LDLR^−/−^ hamsters.

### Fat-1 protects against atherosclerosis in WT and LDLR^−/−^ hamsters under different nutrient conditions

**Fig. 8.**
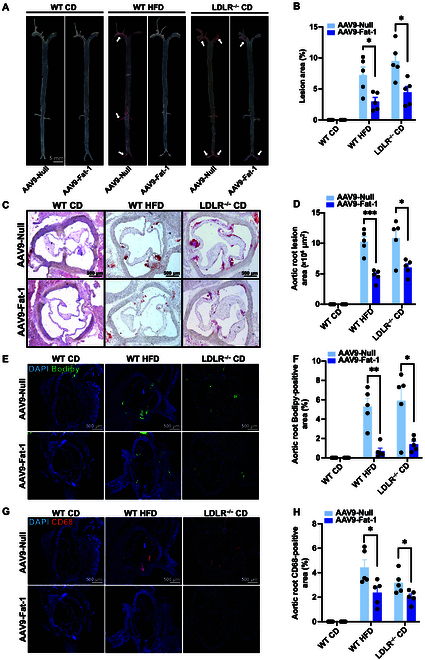
Fat-1 protects against atherosclerosis in WT and LDLR^−/−^ hamsters under different nutrient conditions. (A) Representative images of the en face of the whole aorta stained with Oil Red O in WT and LDLR^−/−^ hamsters with AAV-Null or AAV-Fat-1 administration on CD or HFD feeding (*n* = 5/group). (B) Quantification of atherosclerotic plaque areas in the whole aorta from (A) (*n* = 5/group). *P < 0.05. (C) Representative images of aortic roots stained with Oil Red O from the animals described in (A). (D) Quantification of atherosclerotic lesion areas in the serial cryo-sections of aortic roots. *P < 0.05 and ***P < 0.001. (E to H) Representative Bodipy (E) and CD68 (G) staining images by immunofluorescence of aortic roots from the animals described in (A). Quantification of Bodipy-positive areas (F) and CD68-positive areas (H) in the aortic roots from (E) and (G), respectively. *P < 0.05 and **P < 0.01. Data are presented as mean ± SEM. Statistical significance was determined by the Student *t* test. *P < 0.05; **P < 0.01; ***P < 0.001.

Based on the beneficial effect of Fat-1 on hyperlipidemia and its key role in hepatic lipid metabolism in our 2 different hamster models, we further investigated whether AAV9-Fat-1 treatment could ameliorate atherosclerosis in WT and LDLR^−/−^ hamsters. At the indicated endpoints of our experiments, we collected the aorta and heart for pathological and histological analysis. Oil Red O staining was performed on the whole aorta to assess atherosclerotic lesions. As shown in Fig. 8A, CD-fed WT hamsters had no visible atherosclerotic lesions on CD, while apparent atherosclerotic plaques were observed in HFD-fed WT hamsters and CD-fed LDLR^−/−^ hamsters. However, administration of AAV9-Fat-1 significantly reduced lesion areas in the 2 conditions (Fig. 8A and B). In agreement with the results of whole aorta, the atherosclerotic lesions in the aortic roots were also significantly reduced in HFD-fed WT hamsters and CD-fed LDLR^−/−^ hamsters treated with AAV9-Fat-1 compared to their corresponding controls (Fig. 8C and D). To further confirm the composition of the atherosclerotic lesions, we performed immunohistochemical staining of aortic root sections and found fewer positive areas with Bodipy and CD68 signals, indicating that Fat-1 decreased lipid deposition and inflammatory cell infiltration in the aortic root of WT and LDLR^−/−^ hamsters under HFD and CD conditions, respectively (Fig. 8E to H). Collectively, these data demonstrate that Fat-1 protects against atherosclerosis in WT and LDLR^−/−^ hamsters under different nutrient conditions.

## Discussion

Due to the complexity and heterogeneity of MAFLD pathogenesis and progression, there has long been a dearth of pharmacological interventions for managing MAFLD [29–31]. Recently, resmetirom received approval from the US Food and Drug Administration as the first medication targeting MASH and liver fibrosis, thereby suggesting that modulating essential receptor activation could potentially serve as a therapeutic approach for MAFLD [32]. Despite the use of n-3 PUFA supplementation having gained popularity for lowering plasma lipid levels to protect against metabolic disorders such as MAFLD and atherosclerosis [7,33,34], the therapeutic effects of n-3 PUFAs remain controversial. Independent population-based clinical trials have reported elevated plasma lipids with fish oil administration in individuals with severe hyperlipidemia [35,36]. Studies using different animal species showed that DHA supplementation in hamsters resulted in an increase in LDL-C levels and plasma LDL-C level was elevated in ApoE^−/−^ mice after Fat-1 transgene [10,25], highlighting the prevailing uncertainty surrounding the role of n-3 PUFA supplementation and Fat-1 transgene in lipid metabolism and necessitating further investigation into their application conditions as well as evaluation of their efficacy and safety.

In this study, we constructed an AAV9 vector expressing Fat-1, which was applied to WT and LDLR^−/−^ hamsters to mimic transgene conditions, and then aimed to investigate the impacts of Fat-1 on blood lipids, MAFLD, and atherosclerosis under physiological and/or pathophysiological conditions and explore the possibility that AAV9-based gene therapy of Fat-1 would be considered for the treatment of metabolic disorders. We demonstrated that the expression of Fat-1 led to a substantial reduction in plasma TG, TC, and non-HDL-C levels, accompanied by an increase in HDL-C levels, in both CD- and HFD-fed WT hamsters when compared to their controls that received AAV9-Null, respectively. A similar pattern of observations was also noted in CD-fed LDLR^−/−^ hamsters.

In addition, Fat-1 maintained liver homeostasis in both WT and LDLR^−/−^ hamsters under CD conditions while significantly ameliorating MAFLD induced by HFD in WT hamsters. Further lipidomic analysis of liver revealed that Fat-1 expression significantly increased n-3 PUFAs, particularly DHA, in the livers of WT hamsters under HFD conditions. In LDLR^−/−^ hamsters, Fat-1 led to elevated levels of EPA and stearidonic acid under CD conditions. Recent studies have indicated that these PUFAs can generate bioactive lipids (oxylipins), with their levels in the liver being a subject of particular interest. As biological activity signals, oxylipins can be divided into eicosanoids, specialized pro-resolving lipid mediator specificity (SPMs), and endocannabinoids, which can effectively regulate metabolism and energy homeostasis, inflammation, and tissue repair by binding to specific receptors [37]. Consistently, increased levels of anti-inflammatory lipids such as HDHA and EDP were observed in the livers of HFD-WT hamsters. HDHA is an intermediate of DHA to produce SPMs [38], while 19,20-EDP inhibits liver fibrosis and has cardiovascular protective effects [12], both of which may contribute to the reduction of MAFLD and inflammation. In CD-fed LDLR^−/−^ hamsters, expression of Fat-1 significantly elevated the anti-inflammatory mediators, including EPA-derived HEPE and DHA-derived MaR, while concurrently decreasing the AA-derived pro-inflammatory mediator hydroxyeicosatetraenoic acid, further supporting the beneficial impact of elevated HEPE on dyslipidemia, atherosclerosis, and MAFLD as previously reported in mice [37,38]. Meanwhile, MaR has also been shown to confer hepatoprotection by inhibiting ER stress and promoting the polarization of anti-inflammatory M2 macrophages [11,39], which may contribute to the protective outcomes of Fat-1 overexpression in our hamster model as well.

It should be noted that many lipid-lowering agents have been applied to treat FH in clinical trials [40]; however, the residual risk of ASCVD is still high, implying that more potent drugs need to be developed for urgent use. Importantly, AAV9-Fat-1 also conferred protection against arteriosclerosis in spontaneous (CD-fed LDLR^−/−^ hamsters) and diet-induced (HFD-fed WT hamsters) atherosclerosis models, evidenced by reductions in plaque lesion size, lipid accumulation, and inflammatory cell infiltration in vascular walls. We speculated that this anti-atherosclerotic property might be attributed to the reduction in blood lipids, improvement in the abnormal lipoprotein profile, elevation of HDL levels, and potential enhancement of PUFA functionality within HDL particles. Although AAV9-Fat-1 treatment may facilitate the reduction of lipid levels in LDLR^−/−^ hamsters fed a CD and induce the formation of anti-atherosclerotic plasma lipoprotein profiles, thus serving as a strategy for the reduction of cardiovascular events in patients with FH, we cannot ignore its lipid-lowering effect in CD-fed WT hamsters with normal lipid levels, implying that the application of Fat-1 expression to treat human metabolic diseases should be carefully considered to avoid any adverse effects.

It is widely acknowledged that fatty acids can activate PPARα to regulate metabolic homeostasis. Zhang et al. [41] discovered that DHA, AA, linolenic acid, and lysophosphatidylcholine 22:4 are 4 endogenous metabolites acting as direct agonists of PPARα. PPARα is a member of the PPAR family and belongs to the class of nuclear receptor ligand-activated transcription factors. Extensive evidence demonstrates altered expression of PPARα during the progression of MAFLD and MASH in both animal models and patients. Notably, hepatic expression of PPARα exhibits an inverse correlation with the degree of steatosis, MASH severity, and fibrosis [42–46]. Global knockout or liver-specific knockout of PPARα promotes MAFLD development, highlighting its potential as a therapeutic target for MAFLD/MASH treatment [47–49]. In agreement with previous findings, our liver lipidomic study revealed that AAV9-Fat-1 can endogenously increase the levels of PUFAs and their biologically active lipids in hamster livers, thereby directly activating hepatic PPARα. Unexpectedly, we did not observe any changes in total PPARα protein levels in vivo and in vitro upon Fat-1 treatment, suggesting that increased endogenous PUFAs had no direct effect on PPARα regulation but facilitated its nuclear localization, leading to activation of hepatic PPARα and significant induction of FAO-related genes *Cyp4a10*, *Acox1*, *Acadm*, and *Fgf21*. As a nuclear transcriptional regulator, PPARα governs fatty acid transport, FAO, and ketone body production by activating target genes within the cell nucleus [50]. Therefore, we concluded that promoting the nuclear localization of PPARα represents a promising strategy for treating MAFLD.

It should be noted that the efficacy of fenofibrate, a first-in-class PPARα agonist, on lipid lowering and MAFLD has been extensively investigated in both humans and experimental animals in the past couple of decades [51,52], suggesting that fenofibrate could be a potential drug candidate for the treatment of MAFLD; however, emerging evidence from different clinical studies has shown that fenofibrate cannot improve the pathogenic degree of MAFLD even though it indeed reduces plasma TGs in patients [53,54], thus limiting its further use and indicating that developing a new drug with the capability of generating a favorable plasma lipid profile and mitigating MAFLD progress is urgently needed [55]. When conducting a head-to-head comparison between Fat-1 and fenofibrate, we found that both Fat-1 and fenofibrate effectively ameliorated HFD-induced hyperlipidemia and fatty liver in WT hamsters. Surprisingly, only Fat-1 treatment resulted in increased plasma HDL-C levels, decreased NEFA levels, and optimized plasma apolipoprotein levels and distribution to a better extent. Furthermore, when compared to the control group, we found that Fat-1 exhibited a more pronounced effect on improving pathological phenotypes in MAFLD, as evidenced by better improvement of pathological scores. These results suggest that AAV9-Fat-1 may be an interesting target for drug development and holds potential for being a more efficacious therapeutic option than fenofibrate for treating hyperlipidemia and MAFLD.

In addition to enhancing FAO mediated by PPARα, the anti-MAFLD effect of Fat-1 is accompanied by a reduction in hepatic lipidogenesis and attenuation of inflammation and fibrosis. MAPKs serve as pivotal components of signaling pathways that transmit extracellular stimuli to cells, regulating the expression of multiple genes [56]. Several independent studies have established a strong correlation between the MAPK and PPAR signaling pathways [57–59]. Emerging evidence suggests that p38 MAPK plays a role in response to PPARα [60]. Both the MAPK and PPARα signaling pathways are crucial components of intracellular signal transduction, regulating lipid metabolism and inflammation within cells [61–63]. Some studies indicate that PPARα is a potential downstream target of MAPK signaling [64,65], while others suggest that it can inhibit the phosphorylation and subsequent activation of certain members of the MAPK signaling cascade [66]. Moreover, in vivo experiments have demonstrated that fenofibrate, a PPARα agonist, reduces c-Jun N-terminal kinase and p38 MAPK phosphorylation [67]. Recently, Li Z et al. [68] found that DHA could inhibit tumor necrosis factor α-induced phosphorylation of p38 MAPK in macrophages, thereby suppressing Lp-PLA2, an independent predictor for cardiovascular events associated with inflammation, ultimately reducing inflammation and providing cardiovascular protection. Herein, it is a high possibility in our case that endogenous PUFAs generated by Fat-1 primarily activate PPARα to effectively suppress the downstream pathways of MAPK and cPLA2 to ameliorate inflammatory response, even though a coexistence between activation of the PPARα pathway and inhibition of p38 MAPK phosphorylation occurs; however, further exploration is needed to determine their mutual regulation in future study.

**Fig. 9.**
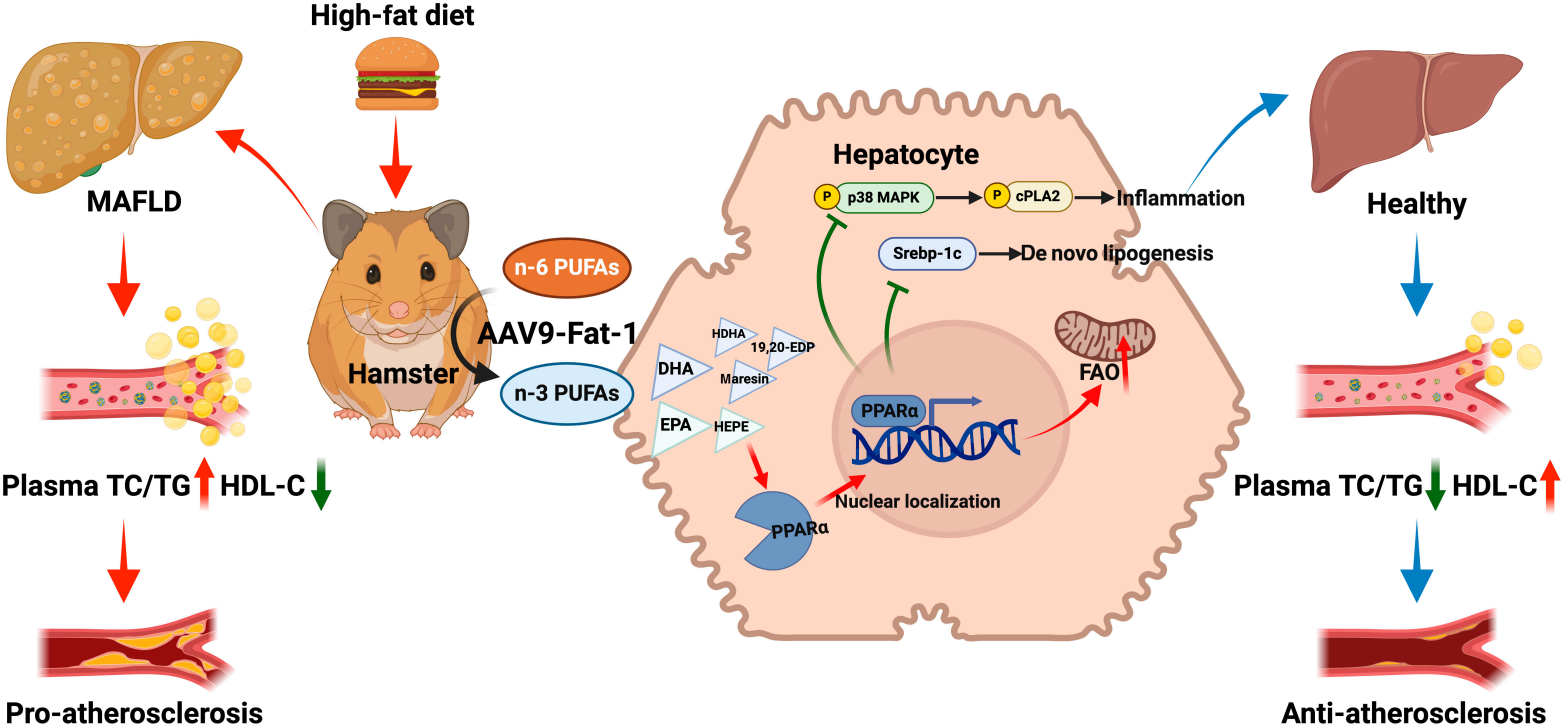
A working model outlining the proposed mechanism. Elevation of endogenous n-3 PUFAs and their derived specialized pro-resolving mediators (SPMs) via jugular vein injection of AAV9-Fat-1 in hamsters. n-3 PUFAs act as signaling molecules and activate the hepatic PPARα signaling pathway by increasing its nuclear localization to facilitate PPARα entry into the nucleus, which enhances fatty acid oxidation and inhibits lipid de novo synthesis and p38 mitogen-activated protein kinase (MAPK)/cPLA2-mediated inflammatory pathways, ultimately mitigating hyperlipidemia, MAFLD, and atherosclerosis. HDHA, hydroxydocosahexaenoic acid; 19,20-EDP, 19,20-epoxydocosapentaenoic acid; FAO, fatty acid β-oxidation.

In summary, this study combined global genomic and metabolomic data from WT and LDLR^−/−^ hamsters expressing AAV9-Fat-1 to establish a novel regulatory network between Fat-1 and PPARα that modulates MAFLD and atherogenesis. Fat-1 directly increases the content of endogenous PUFAs, mainly n-3 PUFAs, as a signal transduction molecule by facilitating the nuclear localization of PPARα to activate the PPARα-mediated signaling pathway, thereby enhancing FAO, inhibiting DNL synthesis, and suppressing inflammation mediated by p38 MAPK/cPLA2 and then ultimately alleviating MAFLD and atherosclerosis development (Fig. 9). These findings provide valuable insights into potential therapeutic strategies for hyperlipidemia, MAFLD, and ASCVD.

## Materials and Methods

The methods for this study’s procedures are outlined in the text and Supplementary Materials. This study includes biological replicates for its datasets, which are available upon request from the corresponding author.

### Construction of AAV9-Fat-1

AAV9-Fat-1 was generated by employing a duplex AAV9 vector carrying the *C. elegans* Fat-1 gene expression frame containing the target sequence of human miR-142-3p (Fig. 1), which was provided by Beijing FivePlus Molecular Medicine Institute. The AAV9-Fat-1 construct has the following novel features: (a) the calmodulin promoter upstream of the Fat-1 gene for tissue-effective expression [69], (b) an optimized Fat-1 coding sequence with a short intron insertion to improve transcription efficiency [70,71], and (c) a safe and nonpathogenic recombinant double-stranded AAV9 vector [72].

### Animals

Our laboratory generated LDLR^−/−^ hamsters using previously described methods [26]. WT Syrian golden hamsters were procured from Vital River Laboratory in Beijing. Both LDLR^−/−^ and WT hamsters were maintained under controlled environmental conditions: 50% to 60% relative humidity, a temperature range of 22 to 24 °C, and a 14-h light/10-h dark cycle. They had free access to water. In all experiments, animals were carefully matched for age and gender. Male hamsters were assigned to receive either a standard CD containing 20% protein and 4% fat (Beijing Ke’ao Company, Beijing, China) or an HFD supplemented with 0.5% cholesterol and 20% fat. WT and LDLR^−/−^ hamsters were injected with AAV9-Null or AAV9-Fat-1 through the intrajugular vein at a dosage of 1 × 10^13^ vg/kg. At the endpoints of the experiments, the animals were humanely anesthetized using 3% pentobarbital sodium, administered at a dosage of 45 mg/kg via intraperitoneal injection.

All experimental procedures were conducted in strict accordance with the guidelines for the care and use of laboratory animals as outlined in National Institutes of Health publication no. 85Y23, revised in 1996. The study was granted approval by the Laboratory Animal Ethics Committee of Peking University, with reference number LA2022147.

### Statistical analysis

All data are presented as mean ± standard error of the mean (SEM). The GraphPad Prism 9.0 software was used for all statistical analysis. Differences between the 2 groups were compared using an unpaired Student *t* test. Multiple-group comparisons were made by one-way analysis of variance (ANOVA) or 2-way ANOVA. Data were considered significant when the *P* value was less than 0.05.

## Data Availability

All data are present in the paper and the Supplementary Materials.
