# Supplementary material for: Fat-1 Ameliorates Metabolic Dysfunction-Associated Fatty Liver Disease and Atherosclerosis through Promoting the Nuclear Localization of PPARα in Hamsters
Source: Research (Wash D C). 2025 Mar 6;8:0577. doi: 10.34133/research.0577 (PMC11884683; doi:10.34133/research.0577)
Supplement: Supplementary 1 — Table S1 Figs. S1 to S3 [file research.0577.f1.docx]

Supplementary Materials for

**Fat-1 ameliorates MAFLD and atherosclerosis through promoting the nuclear localization of PPARα in hamsters**

Wenxi Zhang^1,5^, Jiabao Guo ^1,5^, Guolin Miao^1,5^, Jingxuan Chen^1,5^, Yitong Xu^1^, Pingping Lai^1^, Lianxin Zhang^1^, Yufei Han^1^, Sin Man Lam^3,4^, Guanghou Shui^3^, Yuhui Wang^1^, Wei Huang^1^, Xunde Xian^1, 2,^ **^*^**

**Supplementary method**

**Analysis of plasma lipids and lipoproteins**

Plasma samples from WT and LDLR^-/-^ hamsters were collected after 12h of fasting. Plasma total cholesterol (TC) and triglyceride (TG) levels were measured using commercial enzymatic kits (Biosino Bio Technology & Science, Beijing, China). HDL-cholesterol (HDL-C) level was measured with the same TC kit after ApoB in lipoproteins was precipitated with 20% polyethylene glycol (PEG).

Plasma ApoB, ApoE and ApoA1 levels were measured by Western blotting. Briefly, 1μl of plasma was mixed with 5× SDS loading buffer and heated at 95°C for 10min. The samples were loaded to 6% or 12% sodium dodecyl sulfate polyacrylamide gel (SDS-PAGE) gels, transferred to nitrocellulose (NC) membranes (66485, Pall Bio Trace, USA), blocked with 5% non-fat milk in TBST for 1 hour before incubating with indicated primary antibodies overnight at 4°C. The second antibodies were added for incubation for another 1 hour After washing with 1xTBST. Finally, the protein bands were detected with ECL Kit (Solarbio, PE0010) and visualized in Invitrogen iBright1500 Imaging System (Thermo Fisher, USA). The following antibodies were used: ApoB (178467, Millipore, goat polyclonal IgG, 1:2000), ApoE (178479, Millipore, goat polyclonal IgG, 1:2000) and ApoA1 (ab20453, Abcam, rabbit polyclonal IgG, 1:2000).

Pooled plasma (100μl/group after filtered through a 0.22 mm filter) from hamsters in the indicated group was analyzed for lipid distribution by fast protein liquid chromatography (FPLC, Tricorn high-performance Superose S-6 10/300GL column). The samples were eluted with PBS at a rate of 0.5 mL/min and fractions were collected automatically. The TC and TG levels of the eluted fractions with 500μl of each fraction were measured using the same kit as described above. Three consecutive fractions were mixed with the buffer containing SDS and DTT, and heated at 95 ° C for 10 min. The contents of ApoA1, ApoB, and ApoE were determined by Western blot as described above.

**Plasma biochemical characteristics**

Plasma samples were collected from the retro-orbital plexus of the hamsters after 12h of fasting. Non-esterified fatty acid (NEFA) was measured using commercially enzymatic kit (633-52001, Wako, Japan). The degree of liver injury was evaluated according to plasma alanine aminotransferase (ALT) and aspartate aminotransferase (AST) levels (Nanjing Jiancheng Bioengineering Institute, Nanjing, China).

**Pathological analysis**

Hamsters were sacrificed at indicated time points and 20ml of cold 0.01M PBS was perfused through the left ventricle. Liver, heart and aorta were harvested, fixed overnight in 4% paraformaldehyde (PFA) and then dehydrated in 20% sucrose solution. The liver and heart were embedded in OCT (4583, Sakura, USA), and cryo-sectioned after snap frozen with liquid nitrogen. Hematoxylin-eosin (HE) staining was used to observe morphological changes, oil red O (ORO) staining and Bodipy (D3922, Invitrogen, USA) immunofluorescence staining was used to observe lipid deposition, and Picro sirius red staining was used to observe fibrosis in the sections. Immunofluorescence staining was performed with CD68 antibody (1:200 rabbit polyclonal IgG, BM3639, BOSTER, China) for macrophage in liver. PPARα Immunofluorescence staining was performed with PPARα antibody (1:200, AB227074, Abcam, USA).

**Western blot analysis**

Tissues and cells were lysed with cold RIPA lysis buffer (DiNing, DN105-01, China) containing a protease inhibitor (04693132001; Roche, Basel, BS, Switzerland) and phosphatase inhibitor (4906837001; Roche, Basel, BS, Switzerland). Total protein was extracted by centrifugation and quantified with a BCA kit (23225; Thermo, Waltham, MA, USA). Nuclear and cytoplasmic proteins are extracted using an extraction kit (R0050, Solarbio, China). Samples containing equal quantities of protein were separated by 10% SDS-PAGE, transferred to NC membranes (66485, Pall Bio Trace, USA), blocked with 5% non-fat milk in TBST, incubated with the indicated primary antibodies overnight at 4°C, then followed by the appropriate HRP-conjugated secondary antibodies. Finally, signals were detected with an ECL kit (Transgene, China) and visualized in Invitrogen iBright1500 Imaging System (Thermo Fisher, USA). The antibodies used for our experiments include the PPARα antibody (1:1000, AB227074, Abcam, USA), the LAMINB1 antibody (1:1000, Ab16048, Abcam, USA), the Srebp1c antibody (1:1000, ab28481, Abcam, USA), the Srebp2 antibody (1:1000, ab30682, Abcam, USA), p-p38 (1:1000, TA4001, Abmart, China), p38 (1:1000, T55600, Abmart, China), p-ERK (1:1000, TA1015, Abmart, China), ERK (1:1000, TA0155, Abmart, China), p-JNK (1:1000, 4668, Cell Signaling Technology, USA), JNK (1:1000, 9252, Cell Signaling Technology, USA), p-cPLA2 (1:1000, Ap0968, Abclonal, China), cPLA2 (1:1000, A0394, Abclonal, China), the GAPDH antibody (1:1000, AC002, Abclonal, China). The arbitrary densitometry units of the proteins were quantified by ImageJ image software and expressed as means ± SEM. The results were presented by the ratio of the values normalized to AAV9-Null group.

**Cell culture and *in vitro* treatment**

HepG2 cells were cultured with DMEM supplemented with 10% FBS (FS401, Transgene, China) and 1% penicillin-streptomycin (15140-122, Gibco by Invitrogen, USA) in a 37°C，5% CO_2_ incubator. Plasmid expressing *Caenorhabditis elegans* Fat-1 was transfected into HepG2 cells with Hieff Trans® Liposomal Transfection Reagent (40802ES03, Yeasen, China) to overexpress *Caenorhabditis elegans* Fat-1. In order to inhibit PPARα activity, HepG2 cells were incubated with GW6471 (10μM, HY-15372, MedChemExpress) for 6h, the control group was treated with DMSO (10μM). And then HepG2 cells were exposed to the culture medium containing BSA conjugated-palmitic acid (300μM) (PA; P0500; Sigma-Aldrich, USA) for 12-16 hours. Fatty acid-free BSA (0332, Amresco, USA) was used as a control. After treated with PA, HepG2 cells were washed and collected for subsequent experiments. Immunofluorescence staining of HepG2 cells was performed with Bodipy (D3922, Invitrogen, American) and PPARα antibody (1:200, AB227074, Abcam) after being fixed with 4% paraformaldehyde.

For the nuclear import experiment, HepG2 cells were pretreated with Importazole (IPZ, 10 μM) for 1 h and then transfected with the empty vector (negative control, NC) or plasmid expressing Fat-1. For the nuclear export experiment, HepG2 cells of the same batch were treated with Leptomycin B (LMB, 10 ng/μl) for 24 hours after transfection with NC or plasmid expressing Fat-1.

**RNA isolation and quantitative real time PCR**

Trizol reagent (TransGen, ET111-01-V2) was used to extract total RNA from different tissues. Reverse transcription PCR was performed using a First-Strand cDNA Synthesis Kit (TransGen, AT301-03). Quantitative real-time PCR was performed using primers listed in the Supplementary Table. Amplification reactions were performed using an Mx3000 Multiplex Quantitative PCR System (40 cycles: denaturation at 94°C for 15s, annealing at 60°C for 20s, and extension at 72°C for 45s). GAPDH was used as an internal control for the qPCR.

**Lipid extraction and lipidomics analysis**

Lipids were extracted from approximately 30 mg of frozen tissues using a modified version of the Bligh and Dyer's method as described previously^1^. Briefly, tissues were homogenized in 900 µL of chloroform: methanol: MilliQ H2O (3:6:1) (v/v/v). The homogenate was then incubated at 1500 rpm for 30min at 4°C. At the end of the incubation, 350 µL of deionized water and 300 µL of chloroform were added to induce phase separation. The samples were then centrifuged and the lower organic phase containing lipids was extracted into a clean tube. Lipid extraction was repeated once by adding 500 µL of chloroform to the remaining aqueous phase, and the lipid extracts were pooled into a single tube and dried in the SpeedVac under OH mode. Samples were stored at -80°C until further analysis. The details of lipidomic analysis were showed in supplementary materials.

**Transcriptome analysis**

Total RNA was extracted from liver tissues using TRIzol reagent (ET111-01-V2; Transgene, Beijing, China). After assessment of RNA integrity using the RNA Nano 6000 Assay Kit of the Bioanalyzer 2100 system (Agilent Technologies, CA, USA), the cDNA library was prepared following the Illumina protocol. Then, gene expression profiling was performed in TruSeq PE Cluster Kit v3-cBot-HS (Illumia) by sequencing on an Illumina Novaseq platform, and 150 bp paired-end reads were generated. Next, the clean reads were mapped with Mesocricetus auratus genome using Hisat2 v2.0.5. FPKM (Fragments Per Kilobase of transcript sequence per Millions base pairs) was used to estimate gene expression levels. Differentially expressed genes (DEGs) were identified by differential gene expression analysis conducted with DESeq2. Preparation of RNA library and transcriptome sequencing was conducted by Novogene Co., LTD (Beijing, China). Genes with adjusted p-value < 0.05 and |log (Fold Change) | >1 found by DESeq2 were considered significance. The clusterProfiler R package was used to perform KEGG enrichment analysis of DEGs. The KEGG pathways with padj less than 0.05 were considered significantly enriched by DEGs.

**Drug experiment**

PPARα agonists fenofibrate (Abbott laboratories co. LTD, Shanghai, China) was dissolved in 0.5% sodium carboxymethyl cellulose (CMCNa) and then orally administrated to AAV9-Null-infected hamsters at the dose of 100 mg/kg based on body weight. PPARα antagonist GW6471 (HY-15372, MedChemExpress) was dissolved into 0.5% CMCNa as a stock solution at 5mg/ml and then given to AAV9-Fat-1-infected hamsters at a dose of 1 mg/kg/day via gavage. For AAV9-Null or AAV9-Fat-1 groups, animals received identical volume of 0.5% CMCNa as vehicle solution.

**Plasma glucose analysis**

Plasma glucose was measured with Glucose Assay Kit (BioSino, 100000240), following the manufactures’ protocols.

**GTT and ITT**

GTT was conducted in hamsters after an overnight fast (21:00-9:00), following an intraperitoneal (i.p.) injection of 2g/kg glucose (20% glucose, D-(+)-Glucose, Sigma-Aldrich, G8270) for hamsters. ITT was performed in 4-6 hours fasted hamsters by injecting either 0.75U/kg body weight insulin (Humulin R, Lilly France, HI0219). Plasma glucose levels were determined at 0, 15, 30, 60, and 120 minutes by Glucose Assay Kit in hamsters. Blood samples were taken from hamsters via the intro-orbital vein at the indicated time points.

**Lipid extraction and lipidomics analysis**

Lipidomic analysis of liver was conducted at LipidALL Technologies using a Shimadzu Nexera 20-AD/ExionLC-AD coupled with Sciex QTRAP 6500 PLUS as reported previously^2^. The polar lipids were separated by UP-Hb silica gel column (i.d. 150x2.1 mm, 3 μm) and normal phase (NP)-HPLC method. The mobile phase A was chloroform: methanol: ammonium hydroxide at the ratio of 89.5:10:0.5, and the mobile phase B was Chloroform: methanol: ammonium hydroxide: water at the ratio of 55:39:0.5:5.5. MRM targeted quantitative technique was established for comparative analysis of various polar lipids, and lipids were quantified by adding internal standard. d9-PC32:0(16:0/16:0), d9-PC36:1p(18:0p/18:1), d7-PE33:1(15:0/18:1), d9-PE36:1p(18:0p/18:1), d31-PS(d31-16:0/18:1), d7-PA33:1(15:0/18:1), d7-PG33:1(15:0/18:1), d7-PI33:1(15:0/18:1), C17-SL, d5-CL72:8(18:2)4, Cer d18:1/15:0-d7, d9-SM d18:1/18:1, C8-GluCer, C8-GalCer, d3-LacCer d18:1/16:0, Gb3 d18:1/17:0, d7-LPC18:1, d7-LPE18:1, C17-LPI, C17-LPA, C17-LPS, C17-LPG, d17:1 Sph, d17:1 S1P, C14-BMP, d3-16:0-carnitine were obtained from Avanti Polar Lipids. GM3-d18:1/18:0-d3 was purchased from Matreya LLC. Free fatty acids were quantitated using d31-16:0 (Sigma-Aldrich) and d8-20:4 (Cayman Chemicals).

Glycerol lipids including diacylglycerols (DAG) and triacylglycerols (TAG) were quantified using a modified version of reverse phase HPLC/MRM^3^. Separation of neutral lipids were achieved on a Phenomenex Kinetex-C18 column (i.d. 4.6x100 mm, 2.6 µm) using an isocratic mobile phase containing chloroform:methanol:0.1 M ammonium acetate 100:100:4 (v/v/v) at a flow rate of 300 µL for 10 min. Levels of short-, medium-, and long-chain TAGs were calculated by referencing to spiked internal standards of TAG(14:0)3-d5,TAG(16:0)3-d5 and TAG(18:0)3-d5 obtained from CDN isotopes, respectively. DAGs were quantified using d5-DAG17:0/17:0 and d5-DAG18:1/18:1 as internal standards (Avanti Polar Lipids).

Eicosanoids in samples were quantitated at LipidALL Technologies as previously described^4^. Approximately 200 mg of tissues were extracted in a buffer comprising methanol containing 0.1% (w/v) of butylated hydroxytoluene and butylated hydroxyanisole with formic acid and internal standard cocktail added. Samples were vortexed to allow thorough mixing. A fixed amount of ceramic beads pre-cleaned with methanol was then added and the tissue samples were incubated at 1500 rpm for 12 h at 4℃ to ensure efficient extraction of eicosanoids from the tissue matrix. The samples were centrifuged at 4℃ for 10 min at 12000 rpm, and the supernatant was extracted. The extraction was repeated for a second round. The pooled supernatants were enriched for eicosanoids via solid phase extraction (SPE) using Oasis Prime HLB columns (30 mg, Waters, USA) as previously described^5^. The internal standard cocktail contained PGD2-d4, PGE2-d4, PGF2a-d4, 15-deoxy-D12,14-prostaglandin J2-d4, 6-keto-PGF1a-d4, 13,14-dihydro-15-keto prostaglandin D2-d4, 13,14-dihydro-15-keto prostaglandin F2a-d4, thromboxane B2-d4, HETE: 5(S)-hydroxy-eicosatetraenoic acid-d8 , HETE:12(S)-hydroxy-eicosatetraenoic acid-d8, HETE:15(S)-hydroxy-eicosatetraenoic acid-d8, HETE: 20-hydroxy arachidonic acid-d6, EET: 8(9) epoxy-eicosatrienoic acid-d11, EET: 11(12) epoxy-eicosatrienoic acid-d11, EET: 14(15) epoxy-eicosatrienoic acid-d11, DiHOME: 9,10-dihydroxy octadecenoic acid-d4, DiHOME: 12,13-dihydroxy octadecenoic acid-d4, 5-oxo eiosatetranenoic acid-d7, resolvin D1-d5, leukotriene C4-d5, leukotriene E4-d5, leukotriene B4-d4, HODE: 9(S)-hydroxy-octadecadienoic acid-d4, HODE: 13(S)-hydroxy-octadecadienoic acid-d4, ARA-d11, d31-16:0, EOME: 9,10-EOME-d4, EOME: 12,13-EOME-d4, DHET:11,12-DHET-d10, 5-iso prostaglandin F2a VI-d11 in methanol (Cayman chemicals, USA). SPE eluents were transferred to tubes containing 20 µL of ethanol:glycerol 1:1 (v/v) to prevent complete desiccation, and dried under flowing stream of nitrogen gas. The dried extract was re-constituted immediately in 50 µL of water:acetonitrile:formic acid 63:37:0.02 (v/v/v) for mass spectrometric analysis. Eicosanoid analyses were conducted on an Shimadzu 40X3B-UPLC coupled to Sciex QTRAP 6500 Plus (Sciex, USA). Eicosanoids were separated on a Phenomenex Kinetex-C18 column (i.d. 100x2.1 mm, 1.7µm) with mobile phases comprising (A) water:acetonitrile:formic acid 63:37:0.02 (v/v/v) and (B) acetonitrile:isopropanol 1:1 (v/v) as described previously^4^.

Plasma lipidomics analysis were performed by Novogene Co., LTD (Beijing, China). The samples (100 μL) were taken respectively and homogenized with 300 μL of Isopropanol/acetonitrile (1:1) which contained mixed internal standards and centrifuged at 12,000 rpm for 10 min. Finally, the supernatant (2 μL) was injected into the LC-MS/MS system for analysis. An ultra-high performance liquid chromatography coupled to tandem mass spectrometry (UHPLC-MS/MS) system (ExionLCTM AD UHPLC-QTRAP 6500+, AB SCIEX Corp., Boston, MA, USA) was used to quantitate fatty acids in Novogene Co., Ltd. (Beijing, China). Separation was performed on a Waters ACQUITY UPLC BEH C18 column (2.1×100mm, 1.7μm) which was maintained at 50°C. The mobile phase, consisting of 0.05% formic acid in water (solvent A) and Isopropanol/acetonitrile (1:1) (solvent B), was delivered at a flow rate of 0.30 mL/min. The solvent gradient was set as follows: initial 30% B, 1 min; 30-65% B, 2min; 65-100% B, 11 min; 100% B, 13.5 min; 100-30% B, 14 min; 30% B, 15 min. The mass spectrometer was operated in negative multiple reaction mode (MRM) mode. Parameters were as follows: IonSpray Voltage (-4500 V), Curtain Gas (35 psi), Ion Source Temp (550°C), Ion Source Gas of 1 and 2 (60 psi). LC-MS was used to detect the concentration series of standard solution. The ratio of concentration of standard to internal standard as abscissa, and the ratio of peak area of standard to internal standard as ordinate to investigate the linearity of standard solution. The correlation coefficient (r) > 0.99 of each metabolites were the necessary condition. The limit of quantification (LOQ) were determined by the method of signal-to-noise ratio (S/N), which is comparing the signal measured by the standard solution concentration with the blank matrix. Generally, when the S/N = 10:1, the corresponding concentration is the LOQ^6^.

**Supplementary table 1. Primer sequences for RT-qPCR in hamster liver.**

| Hamster primers | Forward | Reverse |
| --- | --- | --- |
| *Fat-1* | CGACGAGTGGTCCTTTGTGA | GCCGTACTGTGTATCGCTCA |
| *Pparα* | CCTCAGGCTACCACTACGGAGT | TGGAAGCGGCAGTATTGGCATT |
| *Acot1* | AATGGCTCTGTGGCTGCTGTT | TCGCTCTTCCAGTTGTGGTCAT |
| *Cpt2* | GAGCGATGCGTTAAAGGCTG | CATCATGTGCTGAAGCTCGC |
| *Cpt1β* | TTGAGTAGTTGCCCAAGGTGC | ACACACCCCTAAGGATGCCA |
| *Cyp4a10* | ACACCATGGCTGATTCCATCC | GTGTCCACCTCAGCACGTAT |
| *Acox1* | CGTCCGTCCCAAGAACTCC | GGGTCGTATGTGGCTGTAGT |
| *Acox2* | CCTTCCTCAGCTTGCCATGA | TTTGAGTAGCCGTGACCACC |
| *Ucp2* | TCTTGCCGACTGAAGGTTCC | GGAACTTCACCGTGGCTGTA |
| *Acadm* | GGAGGCTACGGCTTCAACACAG | AGCGTCTCTTCAGCAGCAACTT |
| *Ehhadh* | TCTCCTCAGTTGGCGTTCTTGG | GTGTCGGCTCGGAATAACCTCT |
| *Acadl* | GGCGGTATTGGTGGAGACTTGT | CACTTGCCTGCCGTCATCTGA |
| *Fgf21* | TCCTTCGGACCCCTATTGGA | GCTGACAAGACACTCAGGCT |
| *CD36* | AACTGTGGACTCATCGCTGG | ACTGTTCATGGCCACTTCGT |
| *Fatp2* | ACCTATTCCGGAGTCGTGGA | AGCAAGGCCAGTTCCATACC |
| *Fatp5* | GTGCCTTTTCACTTGGCTGG | CTTGTCCCCTGGACCTTGTC |
| *Fabp1* | AAGCCTCGTCGCCACCATGA | TGTGTCGCCGTTGAGTTCAGTC |
| *Srebp1c* | GCGGACGCAGTCTGGG | ATGAGCTGGAGCATGTCTTCAAA |
| *Acc* | ACACTGGCTGGCTGGACAG | CACACAACTCCCAACATGGTG |
| *Fas* | GCAGTCTTGAGTAGCTTTGTGCT | GGGAGCTGTCCAGATTAATACCT |
| *Scd1* | GGAGAAGCAGAAGACCGTTCC | CCCCTCCTCATCCTGGTAGC |
| *Elovl6* | TCTGATGAACAAGCGAGCGA | GACAGGTAAGAGGGAAGGGGT |
| *Fads* | TGGATGCTTGTGCTCACACT | ACAAACAGGGAGGGTAGGGT |
| *CD68* | ACCACCTCCACCCTCTCCAAGT | GTGAGCCGCCCATAAGGAAACG |
| *Adgre1* | CCTGCTATGTCGTGCTGTTCGT | GGCTGTCTGGTTGTCCGTCTTG |
| *IL-1β* | CGGCAGGTGGTGTCAGTCATTG | GGTCAGACAGCACGAGGCATTT |
| *IL6* | AGCCCACCAGGAACGAAAGACA | ACCAGCAGCAGTCCCAAGAAGA |
| *Tnfα* | TCCTGGCCTCCTTTTTGCTT | CCCGTAGGGCGATTACAGTC |
| *IL10* | ACCTGGTAGAAGTGATGCCC | AGTTGCCTCCTGAGGGTCTT |
| *α-SMA* | CCACCATGTACCCAGGCATT | GGCGCTGAACCACAAAACAT |
| *Tgfβ* | CAGTTGTACGGCAGTGGCTGAA | GTCACGGATGGTGCTCATGTCA |
| *Timp1* | CCGCAGCGAGGAGTTTCTCATC | CTGTGGATTCCGTGGCAAGCA |
| *Col1a1* | ATGCCGTGACCTCAAGATGTGC | TGCTCTCGCCGAACCAGACA |
| *Mmp9* | CTCTACACGGAGCACGGCAATG | AACCATCCGAGCGACCTTCAGT |

**Supplementary figures and figure legends**


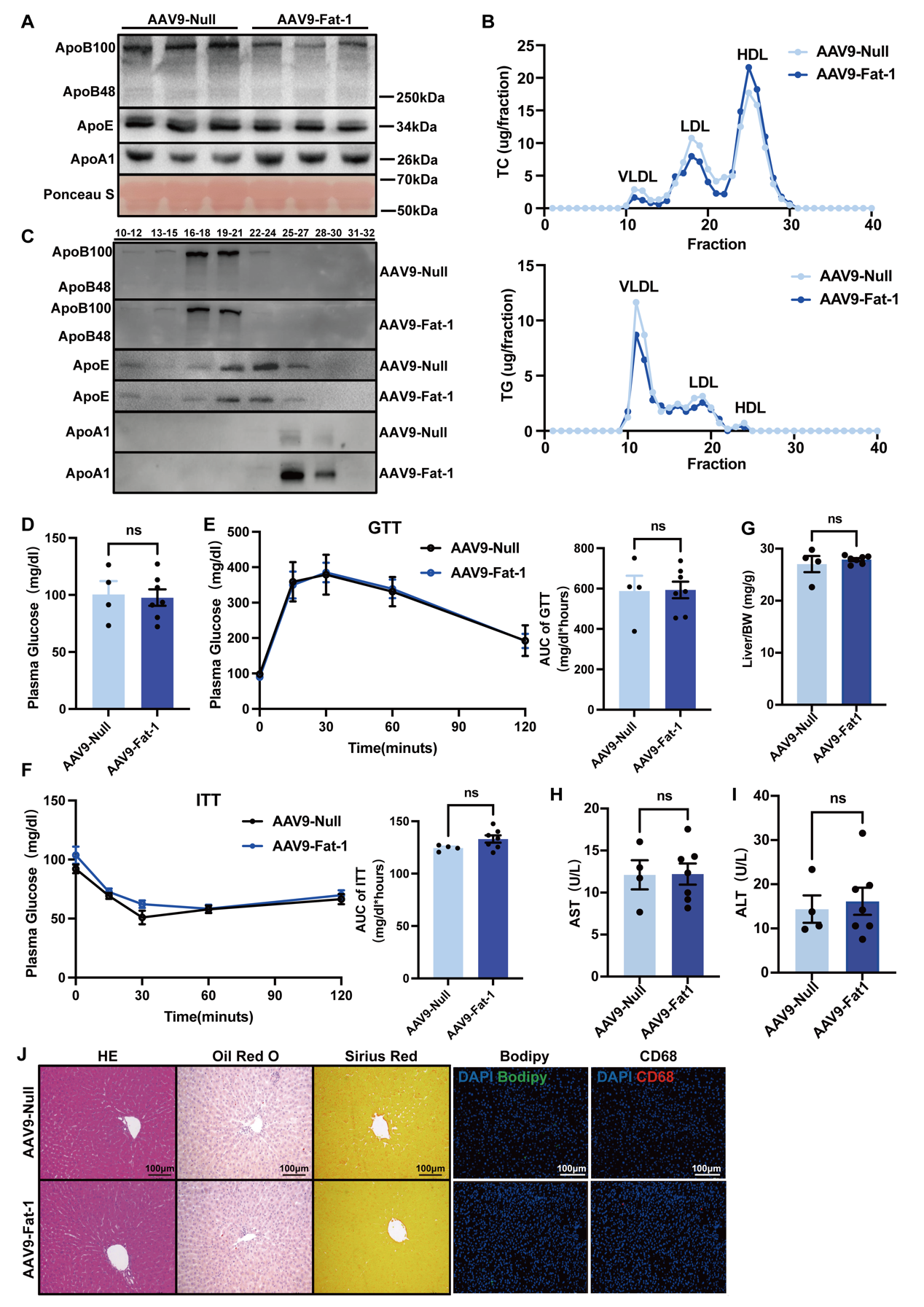


**Figure S1. Fat-1 generates favorable plasma lipid profiles and maintains hepatic homeostasis in CD-fed WT hamsters.**

**A**: Representative images of Western blots showing plasma apolipoproteins (ApoB, ApoE and ApoA1) from CD-fed WT hamsters with or without Fat-1 expression.

**B**: FPLC analysis of TG and TC distribution from pooled plasma in CD-fed AAV9-Null- and AAV9-Fat-1-treatedWT hamsters (n=4-7group).

**C**: Representative Western blots of ApoB, ApoE and ApoA1 in different fractions described in (B).

**D:** Plasma glucose was determined from the CD-fed hamsters (n=4-7/group).

**E:** GTT experiment (2g/kg glucose via i.p. injection) and AUC quantification (n=4-7/group).

**F:** ITT was performed by i.p. insulin injection (0.75U/kg) and AUC quantification for ITT (n=4-7/group).

**G**: The ratio of liver weight and body weight from CD-fed WT hamsters after treatment with AAV9-Null or AAV9-Fat-1 (n=4-7/group).

**H-I**: Plasma AST (H) and ALT (I) were determined from the CD-fed hamsters (n=4-7/group).

**J**: The representative images of HE, Oil red O, and Sirius red staining; Bodipy and CD68 staining by immunofluorescence of the liver sections from the animals described in (G) (n=4-7/group).

ApoB indicates apolipoprotein B; ApoE, apolipoprotein E; ApoA1, apolipoprotein A1; LDL, low-density lipoprotein; VLDL, very-low-density lipoprotein; and FPLC, fast protein liquid chromatography. Data are presented as mean±SEM. Statistical significance was determined by Student’s t test. ns, not significant.

**Supplementary figure2**

**
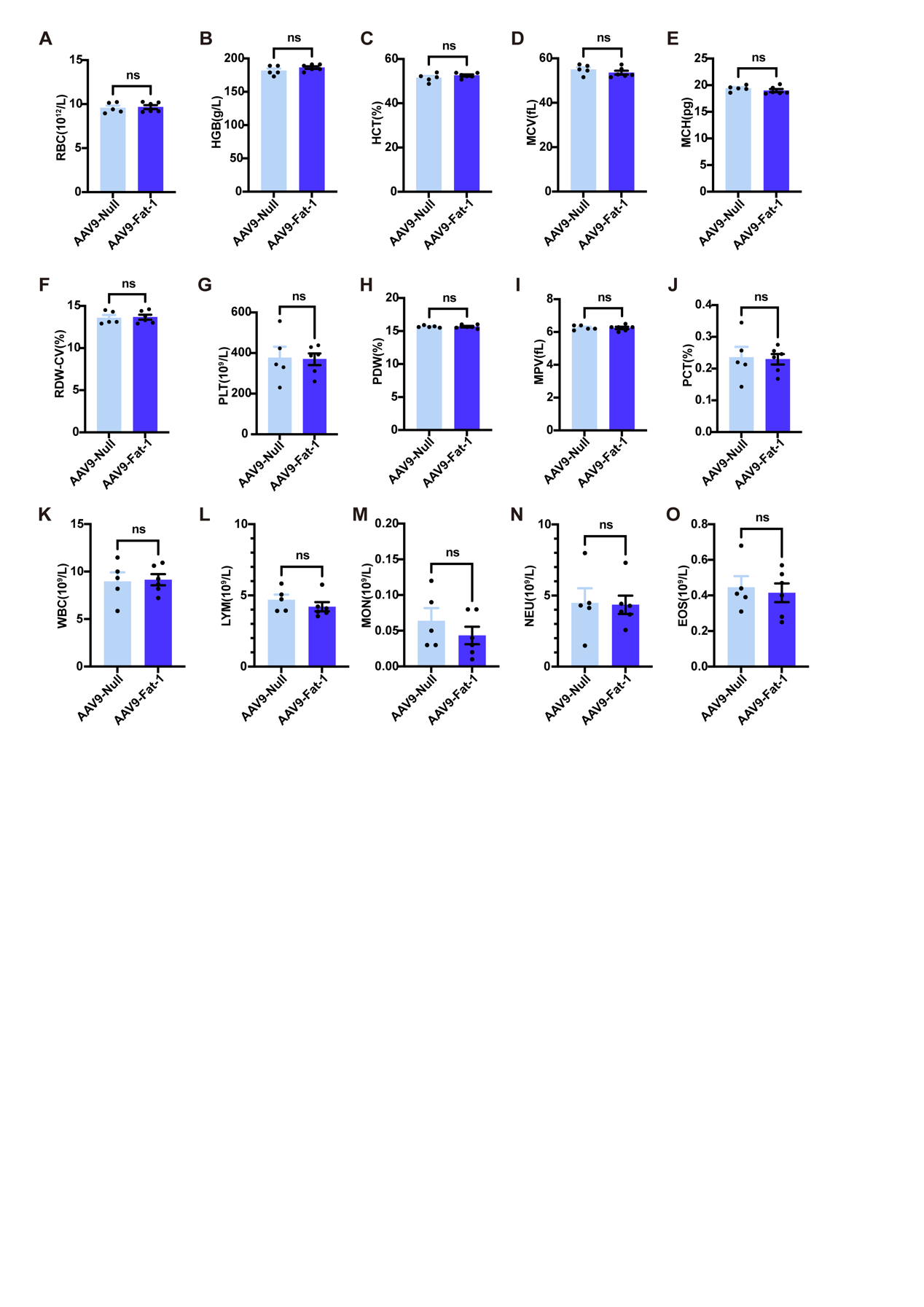
**

**Figure S2. Fat-1 has no effect on blood biochemical parameters in HFD-fed WT hamsters**

**A-O**: The levels of plasma RBC (A), HGB (B), HCT (C), MCV (D), MCH (E), RDW-CV (F), PLT (G), PDW (H), MPV (I), PCT (J), WBC (K), LYM (L), MON (M), NEU (N) and EOS (O) were determined from HFD-fed WT hamsters administrated with AAV9-Null (n=5) or AAV9-Fat-1 (n=6). RBC, red blood cell; HGB, hemoglobin; HCT, hematocrit; MCV, mean corpuscular volume; MCH, mean corpuscular hemoglobin; RDW-CV, red cell distribution width coefficient of variation; PLT, platelets; PDW, platelet distribution width; MPV, mean platelet volume; PCT, procalcitonin; WBC, white blood cell; LYM, lymphocyte; MON, monocyte; NEU, neutrophil; EOS, eosinophil. Data are presented as mean±SEM. Statistical significance was determined by Student’s t test. ns, not significant.

**Supplementary figure
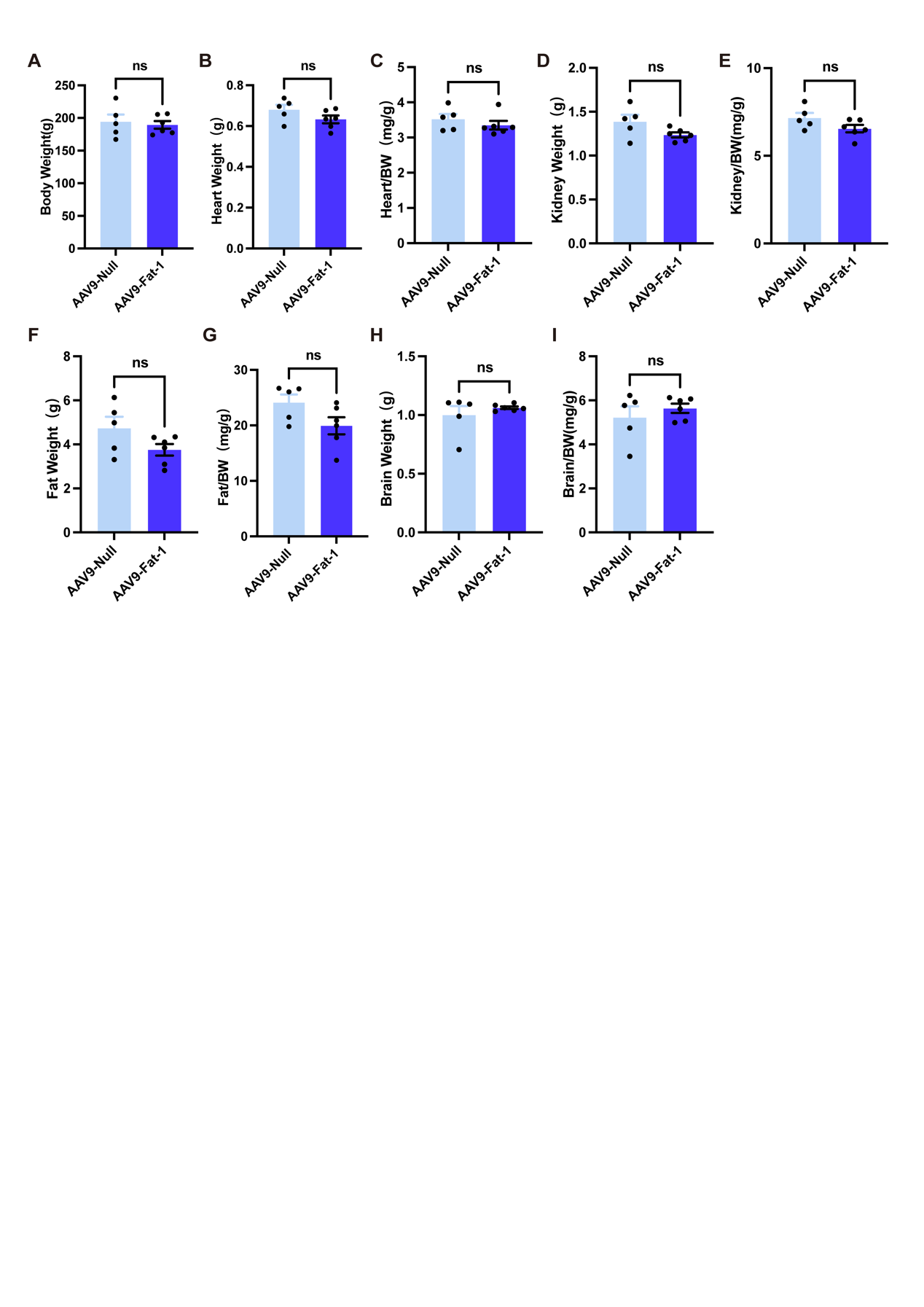
3**

**Figure S3. The administration of Fat-1 has no effect on the weight of other organs in HFD-fed WT hamsters**

**A-I**: Body weight (A), heart weight (B), kidney weight (D), fat weight (F) and brain weight (H) of HFD-fed WT hamsters administrated with AAV9-Null (n=5) or AAV9-Fat-1 (n=6). And the corresponding organ weight ratios (C, E, G, I). Data are presented as mean±SEM. Statistical significance was determined by Student’s t test. ns, not significant.

**References**

**1.** Miao H Li B, Wang Z, Mu J, Tian Y, Jiang B, Zhang S, Gong X, Shui G, Lam SM. Lipidome Atlas of the Developing Heart Uncovers Dynamic Membrane Lipid Attributes Underlying Cardiac Structural and Metabolic Maturation. *Research.* 2022. DOI:<https://doi.org/10.34133/research.0006>

**2.** Lam Sin Man, Zhang Chao, Wang Zehua, Ni Zhen, Zhang Shaohua, Yang Siyuan, Huang Xiahe, Mo Lesong, Li Jie, Lee Bernett, et al. A multi-omics investigation of the composition and function of extracellular vesicles along the temporal trajectory of COVID-19. *Nature Metabolism.* 2021;3(7):909-922. DOI:10.1038/s42255-021-00425-4

**3.** Shui Guanghou, Guan Xue Li, Low Choon Pei, Chua Gek Huey, Goh Joyce Sze Yuin, Yang Hongyuan, Wenk Markus R. Toward one step analysis of cellular lipidomes using liquid chromatography coupled with mass spectrometry: application to Saccharomyces cerevisiae and Schizosaccharomyces pombe lipidomics. *Molecular BioSystems.* 2010;6(6):1008-1017. DOI:10.1039/b913353d

**4.** Lam Sin Man, Wang Zehua, Li Jie, Huang Xun, Shui Guanghou. Sequestration of polyunsaturated fatty acids in membrane phospholipids of Caenorhabditis elegans dauer larva attenuates eicosanoid biosynthesis for prolonged survival. *Redox Biology.* 2017;12:967-977. DOI:10.1016/j.redox.2017.05.002

**5.** Yue Hongfei, Jansen Susan A., Strauss Kenneth I., Borenstein Michael R., Barbe Mary F., Rossi Luella J., Murphy Elise. A liquid chromatography/mass spectrometric method for simultaneous analysis of arachidonic acid and its endogenous eicosanoid metabolites prostaglandins, dihydroxyeicosatrienoic acids, hydroxyeicosatetraenoic acids, and epoxyeicosatrienoic acids in rat brain tissue. *Journal of Pharmaceutical and Biomedical Analysis.* 2007;43(3):1122-1134. <https://pubmed.ncbi.nlm.nih.gov/17125954>

**6.** Chevolleau S., Noguer-Meireles M. H., Jouanin I., Naud N., Pierre F., Gueraud F., Debrauwer L. Development and validation of an ultra high performance liquid chromatography-electrospray tandem mass spectrometry method using selective derivatisation, for the quantification of two reactive aldehydes produced by lipid peroxidation, HNE (4-hydroxy-2(E)-nonenal) and HHE (4-hydroxy-2(E)-hexenal) in faecal water. *Journal of Chromatography. B, Analytical Technologies In the Biomedical and Life Sciences.* 2018;1083:171-179. DOI:10.1016/j.jchromb.2018.03.002
